# AAV-mediated inhibition of ULK1 promotes axonal regeneration in the central nervous system in vitro and in vivo

**DOI:** 10.1038/s41419-021-03503-3

**Published:** 2021-02-26

**Authors:** Vinicius Toledo Ribas, Björn Friedhelm Vahsen, Lars Tatenhorst, Veronica Estrada, Vivian Dambeck, Raquel Alves Almeida, Mathias Bähr, Uwe Michel, Jan Christoph Koch, Hans Werner Müller, Paul Lingor

**Affiliations:** 1 Department of Morphology, Universidade Federal de Minas Gerais, Avenida Presidente Antônio Carlos, 6627, Belo Horizonte, 31270-901 Brazil; 2 Department of Neurology, University Medical Center Göttingen, Robert-Koch-Straße 40, 37075 Göttingen, Germany; 3 Center for Biostructural Imaging of Neurodegeneration (BIN), University Medical Center Göttingen, Von-Siebold-Straße 3a, 37075 Göttingen, Germany; 4 DFG Cluster of Excellence Nanoscale Microscopy and Molecular Physiology of the Brain (CNMPB), University Medical Center Göttingen, Robert-Koch-Straße 40, 37075 Göttingen, Germany; 5 Molecular Neurobiology Laboratory, Department of Neurology, Heinrich-Heine-University Medical Center Düsseldorf, Moorenstraße 5, 40225 Düsseldorf, Germany; 6 Department of Neurology, Rechts der Isar Hospital of the Technical University Munich, Ismaninger Straße 22, 81675 Munich, Germany

**Keywords:** Cell death in the nervous system, Neurodegeneration, Spinal cord injury

## Abstract

Axonal damage is an early step in traumatic and neurodegenerative disorders of the central nervous system (CNS). Damaged axons are not able to regenerate sufficiently in the adult mammalian CNS, leading to permanent neurological deficits. Recently, we showed that inhibition of the autophagic protein ULK1 promotes neuroprotection in different models of neurodegeneration. Moreover, we demonstrated previously that axonal protection improves regeneration of lesioned axons. However, whether axonal protection mediated by ULK1 inhibition could also improve axonal regeneration is unknown. Here, we used an adeno-associated viral (AAV) vector to express a dominant-negative form of ULK1 (AAV.ULK1.DN) and investigated its effects on axonal regeneration in the CNS. We show that AAV.ULK1.DN fosters axonal regeneration and enhances neurite outgrowth in vitro. In addition, AAV.ULK1.DN increases neuronal survival and enhances axonal regeneration after optic nerve lesion, and promotes long-term axonal protection after spinal cord injury (SCI) in vivo. Interestingly, AAV.ULK1.DN also increases serotonergic and dopaminergic axon sprouting after SCI. Mechanistically, AAV.ULK1.DN leads to increased ERK1 activation and reduced expression of RhoA and ROCK2. Our findings outline ULK1 as a key regulator of axonal degeneration and regeneration, and define ULK1 as a promising target to promote neuroprotection and regeneration in the CNS.

## Introduction

Axonal damage is an early event in the course of traumatic injuries to the central nervous system (CNS) and is of major pathophysiological relevance in different chronic neurodegenerative diseases, including Parkinson’s disease, amyotrophic lateral sclerosis, Alzheimer’s disease, and glaucoma^1^. Many of these disorders show a “dying back” degeneration pattern that frequently precedes somatic cell death^2^. Unfortunately, regeneration of damaged axons is severely hampered by extrinsic and intrinsic factors in the adult mammalian CNS^3,4^. Extrinsic factors such as chondroitin sulfate proteoglycans (CSPGs) are presented by glial cells, which confer no-go signals to growth cones and, in combination with the downregulation of intrinsic growth pathways, suppress axonal regeneration. Axon damage therefore results in the loss of function of affected neuronal populations and cannot be sufficiently repaired by current therapies. For instance, after traumatic spinal cord injuries (SCI), the axons in the spinal cord are disconnected from their targets, leading to permanent sensory and motor disabilities^5^. Functional recovery after CNS damage might be improved by prevention or attenuation of axonal degeneration, coupled with the promotion of axonal regeneration. Although various signaling pathways involved in axonal degeneration and regeneration have been identified, the mechanisms governing these events are not fully understood.

In a previous study, we showed an early intra-axonal increase of multiple autophagic proteins, including Unc-51 like autophagy activating kinase 1 (ULK1), autophagy-related (ATG) 7, ATG5, and microtubule-associated proteins 1A/1B light chain 3B (LC3) following SCI, suggesting a possible role of macroautophagy (here: autophagy) in axonal degeneration^6^. Autophagy is an evolutionarily conserved machinery of lysosome-dependent degradation of intracellular cargo^7^. A number of studies suggest that autophagy dysregulation may contribute to a broad spectrum of human diseases, including neurodegenerative disorders^8–10^. Autophagy is coordinated by activation of the ULK1 kinase, the downregulation of which promoted neurite outgrowth of primary neurons in culture^11,12^. Recently, we demonstrated that adeno-associated virus (AAV) vector-mediated overexpression of a dominant-negative form of ULK1 (ULK1.DN) attenuated neurodegeneration in a mouse model of Parkinson’s disease^13^. Furthermore, we showed that ULK1.DN-mediated ULK1 inhibition promoted axonal protection in different models of traumatic axonal lesion, in vitro *and* in vivo, and that application of the ULK1 inhibitor SBI-0206965 protected axons from degeneration induced by optic nerve crush (ONC) in vivo. Mechanistically, ULK1.DN attenuated autophagy, modulated the differential splicing of degeneration-associated genes and increased translation via an mTOR-mediated mechanism^14^. These findings suggest that ULK1 is a key mediator of axonal degeneration. Since preventing axonal degeneration improves the ability of axons to regenerate past a lesion site^15^, we questioned whether ULK1.DN-mediated axonal protection could improve axonal regeneration.

Here, we employ AAV vector-mediated overexpression of ULK1.DN (AAV.ULK1.DN) and analyze the long-term effects on axonal protection and regeneration using in vitro and in vivo models. We show that AAV.ULK1.DN promotes axonal regeneration both in vitro and in a rat model of ONC, and leads to increased neuron survival after optic nerve transection. In addition, AAV.ULK1.DN promotes prolonged axonal stabilization of the corticospinal tract (CST) and enhances axon sprouting of tyrosine hydroxylase (TH) and serotonergic (5-HT) fibers after SCI in vivo. These effects are linked to increased activation of the growth-enhancing kinase ERK1 and decreased levels of the growth suppressors RhoA and ROCK2 after transduction with AAV.ULK1.DN.

## Results

### AAV.ULK1.DN enhances axonal regeneration after axotomy in vitro

We previously generated and characterized an AAV vector expressing ULK1.DN (AAV.ULK1.DN), resulting in the reduction of endogenous ULK1 levels by ~50%, and analyzed the effects of ULK1 inhibition on neuroprotection^13,14^. AAV.ULK1.DN also expresses the fluorescent protein mCherry, which is used to visualize transduced neurons. As control, we produced an almost identical AAV vector expressing mCherry, which however expresses an untranslated 9(5) fragment^16^ instead of ULK1.DN (AAV.CTRL).

**Fig. 1:**
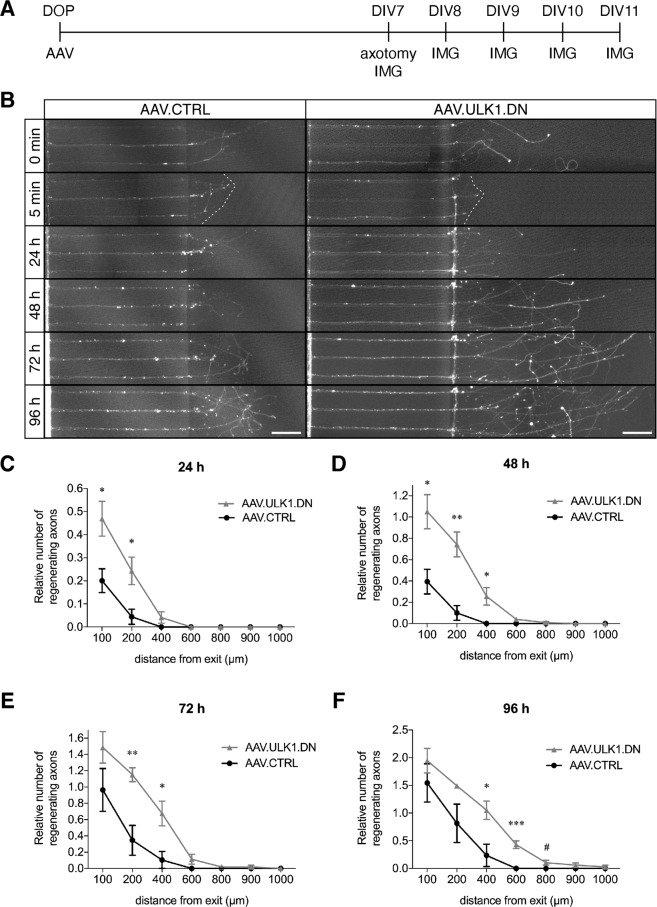
AAV.ULK1.DN enhances axonal regeneration after axotomy in vitro. **A** Scheme of experimental setup for axonal regeneration assays in microfluidic chambers. DOP: day of preparation of E18 rat cortical neurons and seeding in microfluidic culture platforms; DIV: day in vitro; AAV: transduction with adeno-associated viral vectors; IMG: imaging. **B** Representative images of axons growing in microfluidic culture platforms after transduction with viral vectors. Photos were taken directly before axotomy (0 min) to quantify the number of axons before lesion. Images taken 5 min after lesion show successful axotomy (white dashed line). Axons were imaged 24–96 h after axotomy to quantify axon regeneration. Note that for AAV.ULK1.DN, for the 5 min and 24 h time points, the imaged area distal to the longest axon covered a smaller area than the images for the other time points. For visualization purposes, the blank area was filled with gray color. No axons were added or removed. Scale bar: 100 µm. **C**–**F** Quantification of the relative number of regenerating axons (the number of axons at a given time point divided by the number of axons before axotomy) in cells transduced with AAV.CTRL or AAV.ULK1.DN at the indicated distances from the microgroove exit and 24–96 h after axotomy (*n* = 4 independent cultures). Data are presented as means ± SEM; *P < 0.05, **P < 0.01, ***P < 0.001, ^#^*P* = 0.053, according to two-tailed unpaired *t*-test for each distance.

First, we used our previously established paradigm of selective axonal lesions to cultured neurons, in which AAV.ULK1.DN attenuated axonal degeneration over 6 h post injury^14^, to assess a potential pro-regenerative effect of ULK1.DN in vitro. We seeded primary rat cortical neurons in microfluidic culture platforms^17,18^ and applied AAV.ULK1.DN and AAV.CTRL. After 7 days in culture, we performed selective axonal lesions and studied axonal re-growth via live-imaging over 96 h (Fig. 1A). For both viral vectors, we observed re-growth of axons after lesion (Fig. 1B). To quantify axonal regeneration, for each time point (24–96 h), we counted the relative number of regenerating axons (the number of axons at a given time point divided by the number of axons before axotomy) at defined distances (100–1000 µm) from the distal aperture of the microgrooves. Twenty-four hours after axotomy, neurons transduced with AAV.ULK1.DN showed a significantly increased relative number of regenerating axons at 100 µm (46.9% ± 7.6%) and 200 µm distance (24.3% ± 6.0%) compared to AAV.CTRL (100 µm: 20.1% ± 5.2%; 200 µm: 4.4% ± 3.3%) (Fig. 1C). Similarly, 48 h post injury, significantly higher values could be quantified after transduction with AAV.ULK1.DN at 100 µm (105.1% ± 16.0%), 200 µm (74.3% ± 11.7%), and 400 µm distance (25.6% ± 8.2%) compared to AAV.CTRL (100 µm: 39.4% ± 11.6%; 200 µm: 10.1% ± 6.9%; 400 µm: 0.0% ± 0.0%) (Fig. 1D). Seventy-two hours after axotomy, a significant difference in the relative number of regenerating axons was still detectable in cells transduced with AAV.ULK1.DN at 200 µm (115.1% ± 8.5%) and 400 µm distance (67.5% ± 15.1%) compared to AAV.CTRL (200 µm: 34.7% ± 18.3%; 400 µm: 10.4% ± 10.4%) (Fig. 1E). Finally, 96 h post injury, cells transduced with AAV.ULK1.DN showed significantly higher values at 400 µm (105.3% ± 16.6%) and 600 µm distance (42.9% ± 6.9%) as compared with AAV.CTRL (400 µm: 23.7% ± 20.1%, 600 µm: 0.0% ± 0.0%) (Fig. 1F). Furthermore, axons transduced with AAV.ULK1.DN reached up to 1000 µm in length, while AAV.CTRL-transduced axons did not grow beyond 400 µm.

In summary, these data demonstrate that, in addition to attenuated axonal degeneration after lesion, AAV.ULK1.DN also leads to an increased regenerative axon growth over time and length for up to 96 h post injury in vitro.

### AAV.ULK1.DN increases neurite outgrowth on permissive and inhibitory substrate in vitro

**Fig. 2:**
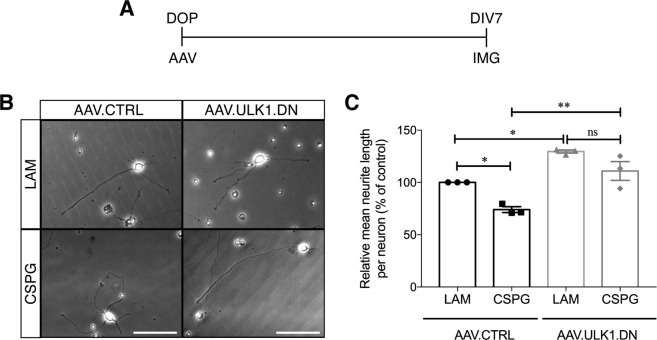
AAV.ULK1.DN increases neurite outgrowth on permissive and non-permissive substrate. **A** Scheme of experimental setup for neurite outgrowth assays. DOP: day of preparation of E18 rat cortical neurons; DIV: day in vitro; AAV: transduction with adeno-associated viral vectors; IMG: imaging. **B** Representative images of cortical neurons cultured on permissive (laminin - LAM) and non-permissive substrate (CSPG) and transduced with viral vectors. Scale bar: 100 µm. **C** Quantification of the relative mean neurite length per cell after transduction with AAV.CTRL and AAV.ULK1.DN (*n* = 3 independent cultures). Data are presented as single data points and means ± SEM; ns = no significant difference, *P < 0.05, **P < 0.01, according to one-way ANOVA and Tukey’s multiple comparisons test.

The finding of increased axonal regeneration after axonal injury in neurons transduced with AAV.ULK1.DN raised the question of whether transduction with AAV.ULK1.DN might also counteract inhibitory environmental cues that are found after axonal injury in the CNS. To assess this, cortical neurons transduced with AAV.ULK1.DN or AAV.CTRL were grown on the permissive substrate laminin as well as the growth-inhibiting matrix CSPG, which is produced by glial cells after CNS lesion^19^. After 7 days in culture, live-imaging was performed to analyze neurite outgrowth (Fig. 2A, B).

Cortical neurons transduced with AAV.CTRL had a mean absolute neurite length per neuron of 564.6 ± 85.9 µm on the permissive substrate laminin, while transduction with AAV.ULK1.DN led to an increase to 729.3 ± 103.7 µm (data not shown). To account for variability in the absolute neurite length per neuron between independent cultures, the relative neurite length per neuron was calculated (i.e., the absolute neurite length per neuron for each condition normalized to the absolute neurite length per neuron of the control condition). Compared to neurons grown on laminin, AAV.CTRL-transduced neurons cultured on the non-permissive substrate CSPG showed a significant reduction in the mean relative neurite length per neuron (74.0 ± 2.8%) (Fig. 2C). In comparison, transduction with AAV.ULK1.DN significantly increased the mean relative neurite length per neuron on both laminin (129.6 ± 1.5%) and CSPG (110.9 ± 9.0%) (Fig. 2C). Taken together, these data demonstrate that transduction with AAV.ULK1.DN significantly enhances neurite outgrowth on both permissive and non-permissive substrate, indicating that inhibition of ULK1 by ULK1.DN is able to foster neurite outgrowth and counteract inhibitory environmental signals in vitro.

### AAV.ULK1.DN fosters axonal regeneration after optic nerve crush

**Fig. 3:**
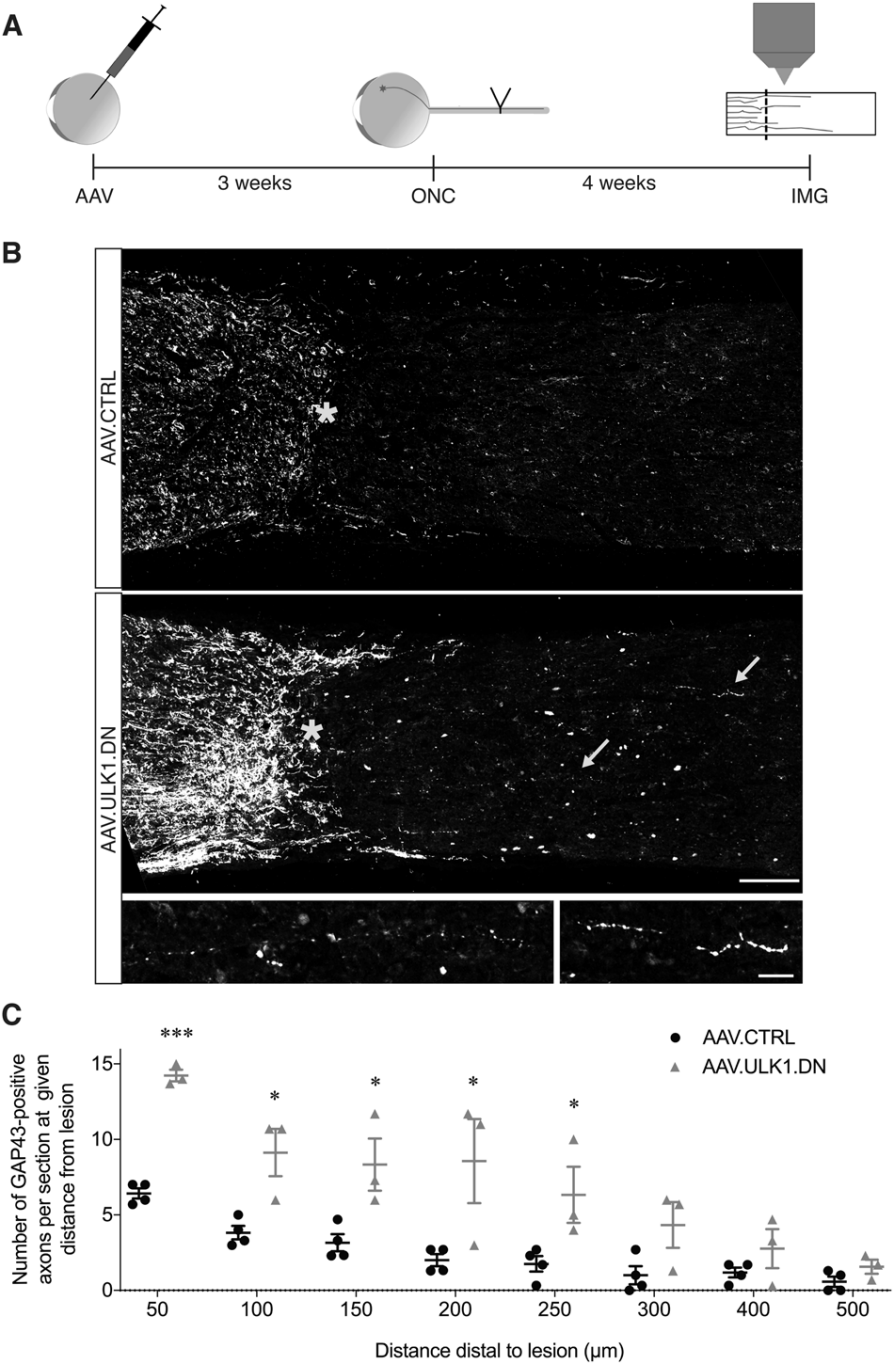
AAV.ULK1.DN enhances axonal regeneration in the optic nerve. **A** Scheme of experimental setup. AAV vectors were intravitreally injected 3 weeks before ONC. Four weeks after ONC, the number of GAP43-positive regenerating axons at different distances from the crush site was quantified on longitudinal optic nerve sections. AAV: transduction with adeno-associated viral vectors; ONC: optic nerve crush; IMG: imaging. **B** Representative images of longitudinal optic nerve sections transduced with given AAV after immunostaining for GAP43. The crush site is marked with an asterisk. The proximal end of the optic nerve is located on the left, the distal end on the right. Insets at the bottom show higher magnification of single GAP43-positive axons distal to the crush site indicated by arrows in the AAV.ULK1.DN group. Scale bar: 100 μm; scale bar inset: 20 µm. **C** Quantification of the number of GAP43-positive axons at different distances distal to the crush site after transduction with AAV.CTRL (*n* = 4 animals) or AAV.ULK1.DN (*n* = 3 animals). Data are presented as single data points and means ± SEM. *P < 0.05, ***P < 0.001, according to two-tailed unpaired *t*-test for each distance.

Since AAV.ULK1.DN improved axonal regeneration and neurite outgrowth in vitro, we evaluated whether AAV.ULK1.DN might also lead to beneficial effects on axonal regeneration in a much more complex environment in vivo. To this end, we used our previously reported experimental paradigm of ONC in vivo^15^. AAV.ULK1.DN and AAV.CTRL were injected intravitreally to label RCG and, 3 weeks later, an ONC was performed as described before^20^. After an additional 4 weeks, axonal regeneration was analyzed by GAP43 immunostaining on longitudinal optic nerve sections (Fig. 3A).

In animals injected with AAV.CTRL, we found very few GAP43-positive axons distal do the crush lesion (Fig. 3B). In contrast, a higher number of GAP43-positive axonal fibers growing beyond the lesion site were observed in animals treated with AAV.ULK1.DN (Fig. 3B). Quantitatively, AAV.ULK1.DN caused a significant increase of two to three fold in the mean number of regenerating axons up to 250 µm past the lesion site compared to AAV.CTRL (Fig. 3C). We also found that in the proximal region of the optic nerve, GAP43 signal is more intense in animals injected with AAV.ULK1.DN, suggesting a persistent axonal protection. Together, these results show that inhibition of ULK1 enhances axon outgrowth in the optic nerve in vivo.

### AAV.ULK1.DN increases RGC survival after optic nerve transection

**Fig. 4:**
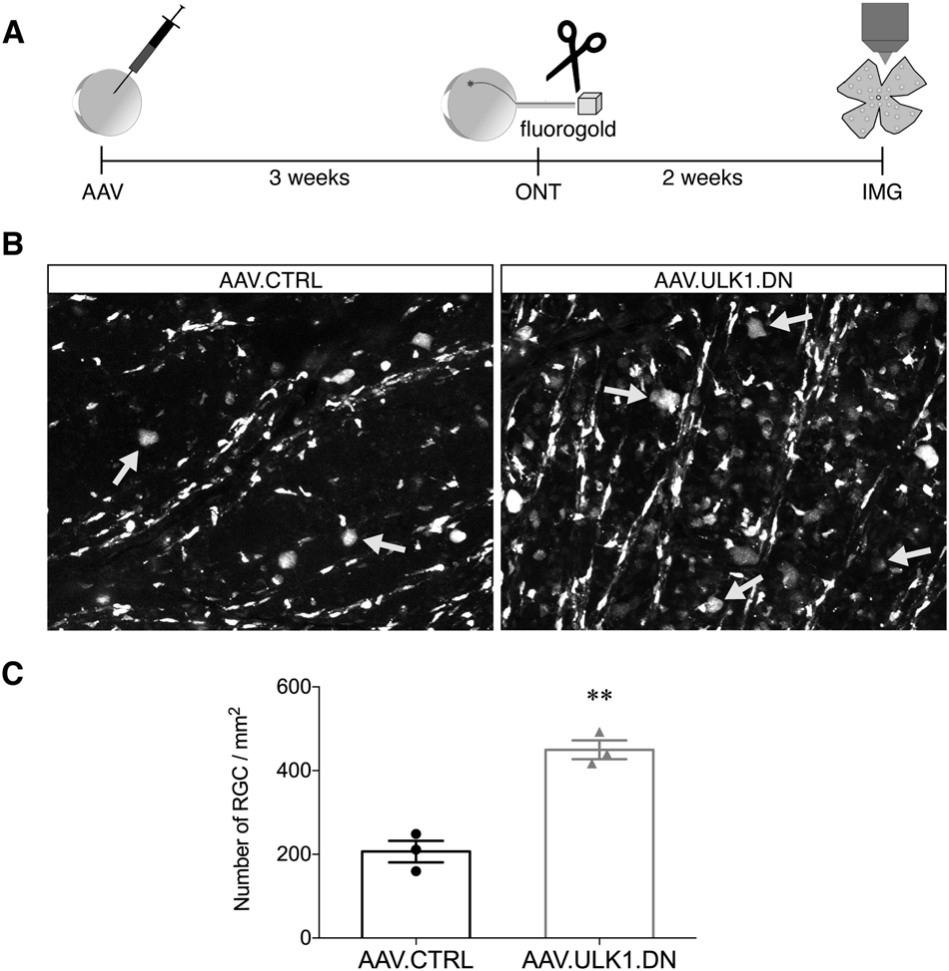
AAV.ULK1.DN increases RGC survival after optic nerve transection. **A** Scheme of experimental setup. AAV vectors were intravitreally injected 3 weeks before optic nerve transection. Immediately after the lesion, RGCs were retrogradely labeled by application of FluoroGold onto the optic nerve stump. Survival of RGCs was evaluated 2 weeks post lesion on retina flat-mounts. AAV: transduction with adeno-associated viral vectors; ONT: optic nerve transection; IMG: imaging. **B** Representative images of the inner retina showing FluoroGold-positive cells in retina flat-mounts transduced with given AAV. Arrows indicate FluoroGold-positive RGCs, identified by morphological criteria. Scale bar: 50 μm. **C** Quantification of the mean number of FluoroGold-positive RGCs in the inner retinal radii after transduction with AAV.CTRL (*n* = 3 animals) or AAV.ULK1.DN (*n* = 3 animals). Data are presented as single data points and means ± SEM. **P < 0.01, according to two-tailed unpaired *t*-test.

We then additionally evaluated whether AAV.ULK1.DN might also enhance neuronal survival after axonal lesion. As before, we injected AAV.ULK1.DN and AAV.CTRL intravitreally. Three weeks later, an optic nerve transection was performed as described previously^21^. For the visualization of cell survival post injury, we retrogradely labeled RGCs by administration of FluoroGold onto the optic nerve stump immediately after the lesion. Two weeks after optic nerve transection, retina flat-mounts were used to quantify the number of FluoroGold-labeled RGCs in the central region of the retina, where virus transduction was observed (Fig. 4A). In animals injected with AAV.CTRL, the mean number of surviving RGC was 207 ± 26 cells/mm^2^ (Fig. 4B, C). Compared to this, transduction with AAV.ULK1.DN resulted in a significant increase in RGC survival, with a mean number of RGCs of 450 ± 22 cells/mm^2^ (Fig. 4C).

### AAV.ULK1.DN promotes long-term axonal protection with no effect on axonal regeneration after spinal cord injury

**Fig. 5:**
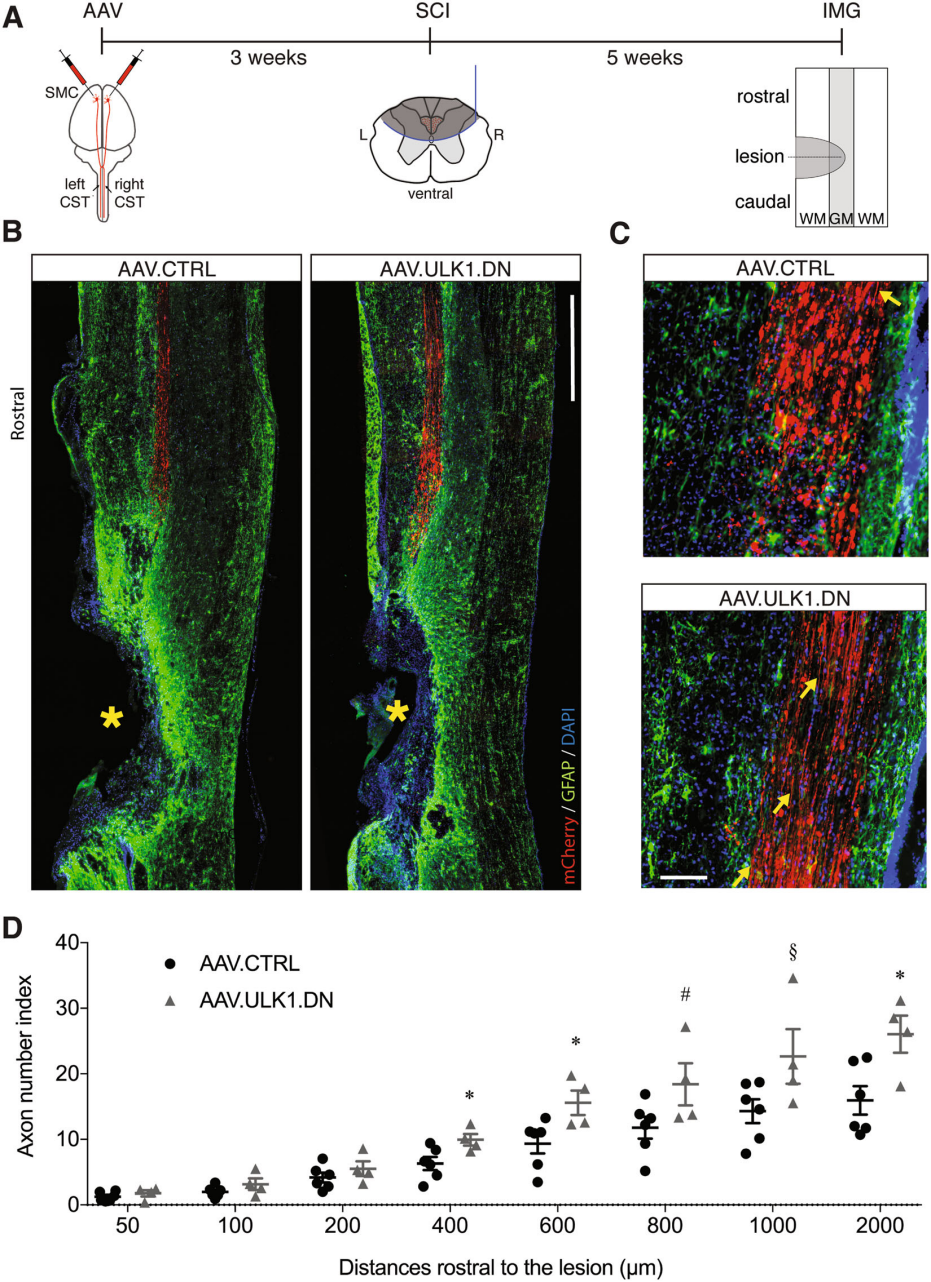
AAV.ULK1.DN mediates long-lasting axonal protection after spinal cord injury. **A** Scheme of experimental setup. AAV vectors were injected into both sensorimotor cortices 3 weeks before SCI. Five weeks after SCI, parasagittal spinal cord sections were obtained. AAV: transduction with adeno-associated viral vectors; SMC: sensorimotor cortex; CST: corticospinal tract; SCI: spinal cord injury; L: left; R: right; IMG: imaging; WM: white matter; GM: gray matter. **B** Representative images of parasagittal spinal cord sections, including the lesion site and rostral region, transduced with given AAV showing mCherry-positive axons (red) and staining for GFAP (green) and DAPI (blue) up to 3000 µm rostral to lesion. Asterisks indicate the lesion areas. Scale bar: 1000 μm. **C** Higher magnification images of areas at around 2000 µm distance from the lesion border. Arrows indicate intact axons. Scale bar: 100 µm. **D** Quantification of the axon number index (a ratio of intact axon numbers to the total number of transduced axons) at the indicated distances from the lesion border after transduction with AAV.CTRL (*n* = 6 animals) and AAV.ULK1.DN (*n* = 4 animals). Data are presented as single data points and means ± SEM. *P < 0.05, ^#^*P* = 0.08, ^§^*P* = 0.07, according to two-tailed unpaired *t*-test for each distance.

Having found increased axonal regeneration by AAV.ULK1.DN in vitro and after ONC in vivo, we next aimed to examine whether ULK1 inhibition might also promote axon re-growth in a rat model of spinal cord injury. To assess this, we injected the AAV vectors into the sensorimotor cortex of both hemispheres to label the CSTs on both sides. Three weeks after viral injections, the spinal cord was partially lesioned with a wire knife, leading to the transection of both dorsal CST as described before^22^. Five weeks later, the animals were euthanized and immunohistochemical staining was performed (Fig. 5A).

For both viral vectors, the transduction rates of CST axons in coronal sections of the cervical spinal cord were similar (Fig. S1A, B). Equally, we observed no differences in the lesion size as assessed by GFAP staining between both vectors (Fig. S2A, B). Furthermore, we did not detect any mCherry-positive axons caudal to the lesion in parasagittal spinal cord sections after transduction with either AAV vector (Fig. 5B), suggesting that AAV.ULK1.DN does not promote axonal regeneration in this model.

In our previous study, we found that AAV.ULK1.DN protected rubrospinal tract axons from degeneration 1 week after SCI^14^. To investigate if this effect was sustained 5 weeks after lesion, we quantified the axon number index (a ratio of intact axon numbers to the total number of transduced axons) at different distances rostral to the lesion border. We found that transduction with AAV.ULK1.DN significantly increased the number of intact axons at 400 µm (from 6.3 ± 0.1 to 9.9 ± 0.9), 600 µm (from 9.4 ± 1.5 to 15.6 ± 1.9) and 2000 µm (from 16.0 ± 2.2 to 26.1 ± 2.8) rostral to the lesion compared to AAV.CTRL (Fig. 5C, D). There was also a trend to increased numbers of intact axons at 800 µm (from 11.8 ± 1.7 to 18.4 ± 3.2) and 1000 µm (from 14.3 ± 1.8 to 22.7 ± 4.2); however, these values were not significantly different (Fig. 5D). Taken together, these results suggest that ULK1 inhibition promotes long-lasting axonal protection but does not modulate axonal regeneration in this SCI model.

### AAV.ULK1.DN stimulates axon sprouting of TH and 5-HT fibers, but not CST fibers, after spinal cord injury

Axon sprouting is a form of plasticity widely described to occur after SCI^23–25^. Specifically, sprouting of TH and 5-HT axons was reported previously in a rat model of SCI^26^. Therefore, we additionally evaluated the distribution of mCherry-positive CST fibers as well as TH- and 5-HT-positive fibers after transduction with both AAV vectors in our model.

**Fig. 6:**
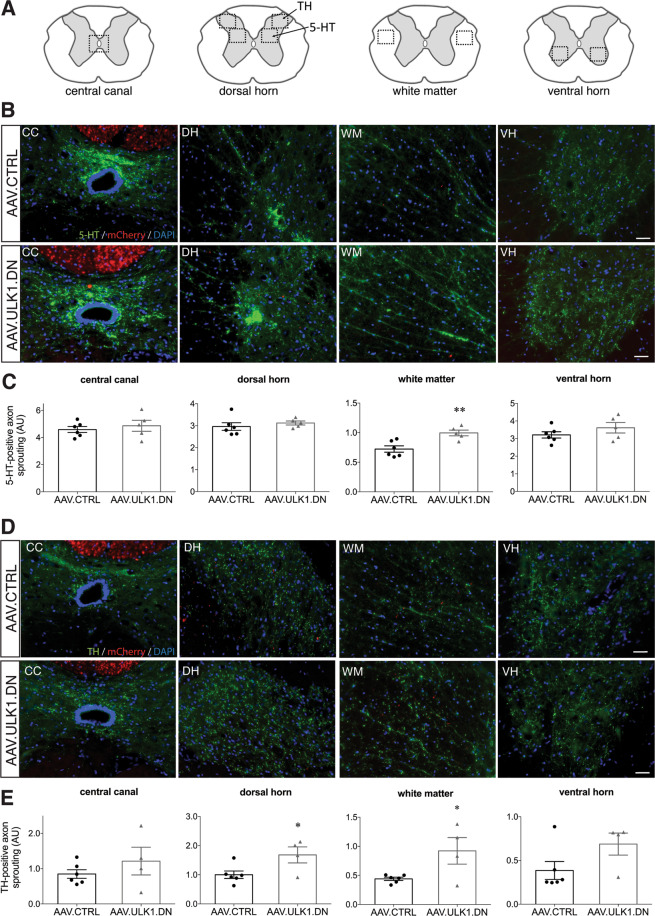
AAV.ULK1.DN promotes axon sprouting after spinal cord injury. **A** Schematic overview of areas used for the quantification of axon sprouting rostral to the lesion at cervical level C2. **B**, **D** Representative images of coronal spinal cord sections (C2) transduced with given AAV showing mCherry-positive axons (red) and staining for 5-HT (**B**-green), TH (**D**-green) and DAPI (blue). CC: central canal; DH: dorsal horn; WM: white matter; VH: ventral horn. Scale bar: 100 μm. **C**, **E** Quantification of 5-HT (**C**) and TH (**E**) axon sprouting after transduction with AAV.CTRL (*n* = 6 animals) and AAV.ULK1.DN (*n* = 4 animals). AU: arbitrary units. Data are presented as single data points and means ± SEM. *P < 0.05, according to two-tailed unpaired *t*-test.

To assess the distribution of mCherry-positive CST sprouts after lesion, the densities of mCherry-labeled axonal sprouts were measured in the white matter of coronal sections rostral to the lesion, at cervical level C2 (Fig. 6A). We found no differences in the axon sprouting of CST fibers in the white matter comparing AAV.ULK1.DN- and AAV.CTRL-transduced animals (Fig. S3A, B). Then, we quantitatively assessed 5-HT- and TH-labeled axon densities around the central canal (CC), in the dorsal horn (DH), white matter (WM) and ventral horn (VH) regions (Fig. 6A). For the 5-HT fiber distribution, we found equal densities in the CC, DH, and VH regions (Fig. 6B, C). In the WM region, however, a significant increase was observed in AAV.ULK1.DN-transduced animals (Fig. 6B, C). The fiber distribution of TH-positive axons was similar for animals injected with AAV.ULK1.DN and AAV.CTRL in the CC and VH regions (Fig. 6D, E). However, in the WM and DH regions, there was a significant increase in TH-positive axonal densities in the AAV.ULK1.DN group compared to AAV.CTRL (Fig. 6D, E). Taken together, these results suggest that cortical expression of ULK1.DN indirectly stimulates axon sprouting of TH- and 5-HT-positive fibers in the SC with no effect on CST fibers after SCI.

### AAV.ULK1.DN exerts pro-regenerative effects via enhanced ERK1 activation and downregulation of ROCK2 and RhoA

We previously demonstrated decreased autophagy, enhanced mTOR-mediated protein translation, and differential splicing of degeneration and regeneration-associated genes by ULK1.DN^14^. To better understand the molecular mechanisms responsible for the observed pro-regenerative effects in this study, we analyzed the expression of additional proteins that are known to be implicated in axonal regeneration in primary rat cortical neuron cultures. To assess whether potential effects of AAV.ULK1.DN are connected to its autophagy-inhibiting function, we applied the autophagy-inducing drug rapamycin in selected conditions.

**Fig. 7:**
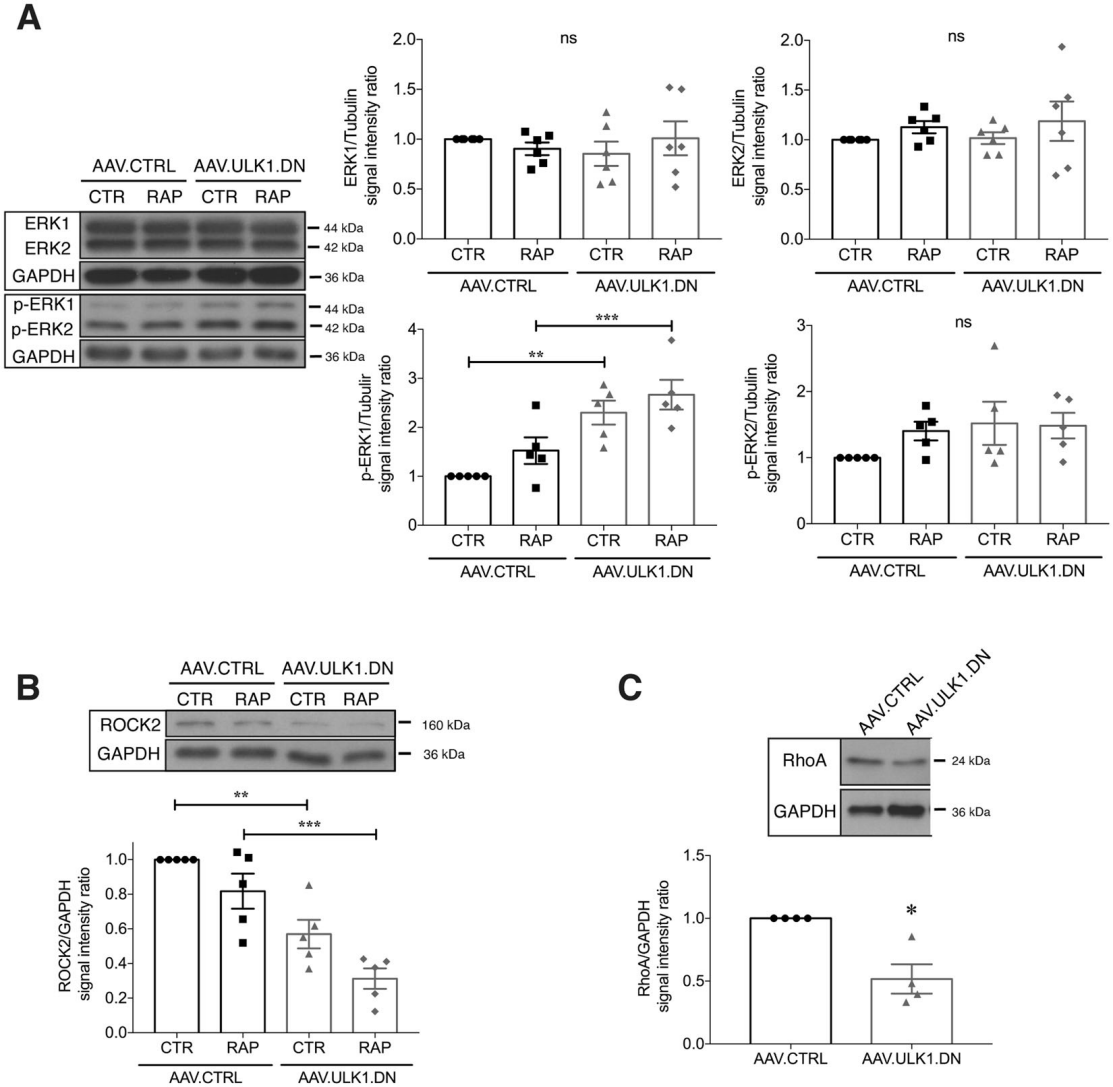
AAV.ULK1.DN alters ERK, ROCK2, and RhoA signaling. Lysates were obtained from E18 rat cortical neurons on DIV 8 after transduction with AAV.ULK1.DN or AAV.mCherry. **A**–**C** Representative immunoblots of ERK1/2 (**A**), p-ERK1/2 (**A**), ROCK2 (**B**), RhoA (**C**) and the corresponding bands of the loading control GAPDH are shown. Quantifications of the band intensities of ERK1/2 (**A**-*n* = 6 independent cultures**)**, p-ERK1/2 (**A**-*n* = 5 independent cultures), ROCK2 (**B**-*n* = 5 independent cultures), and RhoA (**C**-*n* = 4 independent cultures) normalized to GAPDH as loading control. CTR: control. RAP: addition of rapamycin (750 nM) 24 h before lysis. Data are presented as single data points and means ± SEM. *P < 0.05, **P < 0.01, ***P < 0.001, ns: no significant difference, according to one-way ANOVA and Tukey’s multiple comparisons test (**A**, **B**) or one-sample *t*-test (**C**).

First, we investigated the expression of ERK, which is strongly implicated in axonal regeneration^27^. Analysis of the two isoforms ERK1/2 showed similar expression after transduction with AAV.ULK1.DN and AAV.CTRL (Fig. 7A). However, quantification of the phosphorylated forms (p-ERK1/2), which correspond to activated ERK1/2^28^, demonstrated significantly increased p-ERK1 levels upon transduction with AAV.ULK1.DN in naïve and rapamycin-treated culture conditions (Fig. 7A). Rapamycin treatment did not alter p-ERK levels, suggesting that increased p-ERK1 signaling is a molecular mediator of the pro-regenerative effects of ULK1.DN that is independent of its autophagy-modulating function. To evaluate possible downstream targets of ERK, we analyzed the expression of CREB, p-CREB (Fig. S5E), ELK and p-ELK (Fig. S5F). However, no significant differences were detectable between neurons transduced with AAV.ULK1.DN and AAV.CTRL, indicating that ERK exerts its role independent of CREB and ELK.

Next, we analyzed the levels of RhoA and ROCK2, two major regulators of axonal degeneration and regeneration in the CNS^29,30^. Interestingly, we observed significantly lower levels of RhoA and ROCK2 after transduction with AAV.ULK1.DN compared to AAV.CTRL (Fig. 7B, C), indicating that ULK1.DN additionally mediates axonal regeneration via downregulation of RhoA and ROCK2. Again, we observed no effect of rapamycin treatment on ROCK2 levels (Fig. 7B), suggesting that AAV.ULK1.DN also affects ROCK2 expression independent of its autophagy-inhibiting function.

We then studied the expression of several targets of ROCK2, some of which we previously found to be altered after AAV-mediated downregulation of ROCK2^29^. First, we analyzed the levels of the pro-survival factor AKT and its phosphorylated form (p-AKT). AKT showed similar expression after transduction with AAV.CTRL and AAV.ULK1.DN, while p-AKT levels showed a small but significant reduction by ULK1.DN (Fig. S4A), indicating that the pro-regenerative effects observed in this study are independent of AKT signaling. Consistently, the expression levels of PTEN and p-PTEN, an upstream regulator of AKT, were equal between AAV.ULK1.DN and AAV.CTRL (Fig. S4B). Similarly, the expression of CRMP2 and p-CRMP2, a downstream target of ROCK2 and important mediator of axonal outgrowth^47^, were not significantly different between AAV.ULK1.DN and AAV.CTRL (Fig. S4C, D). To assess if reduced ROCK2 levels by AAV.ULK1.DN affect actin dynamics, we quantified the F/G-actin ratio in whole cell lysates, which, however, was not significantly changed (Fig. S4G).

Lastly, we analyzed the expression of additional pathways that play crucial roles in axonal outgrowth and regeneration, including the GSK3β, JNK, and STAT3 pathways. However, we found no differences in the expression of GSK3β, p-GSK3β, p-JNK, and STAT3 between AAV.ULK1.DN and AAV.CTRL, while the levels of phosphorylated (active) STAT3 were significantly lower after transduction with ULK1.DN (Fig. S5A–D). Finally, as we have previously demonstrated that AAV.ULK1.DN leads to the differential exon usage of *Ddit3* and *Kif1b*^14^, we evaluated whether alterations in their protein levels can be detected. Transduction with AAV.ULK1.DN, however, did not change the total protein expression of DDIT3 (Fig. S4E) and KIF1B (Fig. S4F).

Taken together, we demonstrate enhanced p-ERK1 levels as well as reduced expression of RhoA and ROCK2 by ULK1.DN, suggesting that these proteins represent molecular mediators of the pro-regenerative effects of ULK1.DN.

## Discussion

**Fig. 8:**
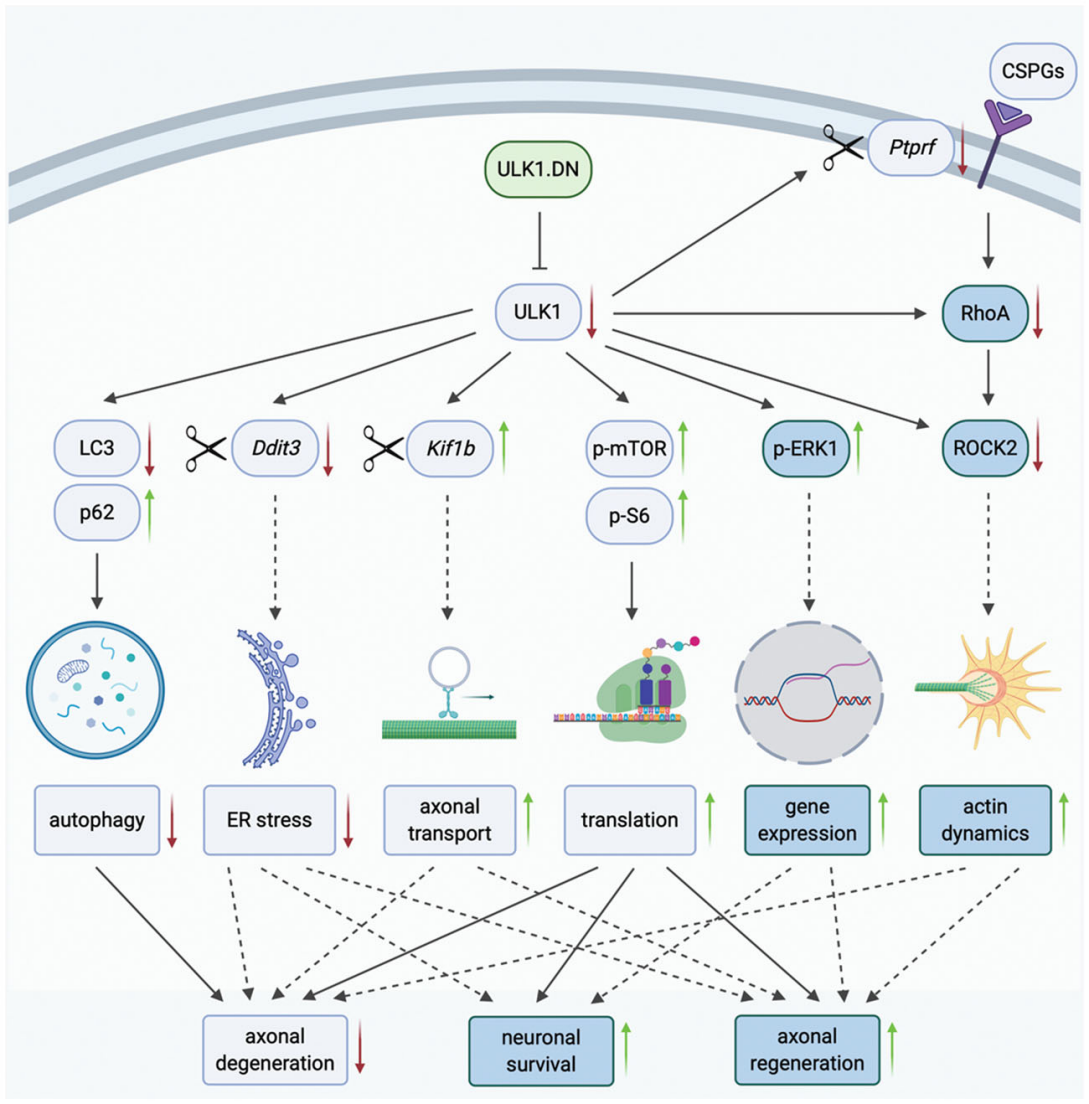
Overview of the molecular mechanisms underlying the effects of AAV.ULK1.DN-mediated ULK1 inhibition on axonal degeneration, neuronal survival, and axonal regeneration. We have previously demonstrated that inhibition of ULK1 function via expression of dominant-negative ULK1 (ULK1.DN) attenuates axonal degeneration in different models of axonal injury in vitro and in vivo^14^. We connected these findings to reduced autophagy, increased mTOR-dependent translation, and differential splicing of the genes *Kif1b* and *Ddit3*, which we hypothesized to enhance axonal transport and reduce ER stress (previous results depicted in light blue). In this study, we additionally demonstrate that ULK1.DN-mediated ULK1 inhibition promotes neuronal survival and fosters axonal regeneration after axonal injury (results of this study depicted in dark blue). Mechanistically, we extend our previous findings by demonstrating elevated levels of p-ERK1 after transduction with AAV.ULK1.DN, which could promote survival and enhance the intrinsic axonal growth capacity via increased gene expression. Furthermore, we show reduced expression of the RhoA-ROCK2 pathway in ULK1.DN-transduced neurons, which counteracts the growth-inhibitory effect of glia-derived molecules such as CSPGs after lesion and could result in increased pro-regenerative actin dynamics after lesion. Correspondingly, ULK1.DN also mediates differential splicing of the CSPG receptor *Ptprf*^14^. Together, we propose that AAV.ULK1.DN-mediated ULK1 inhibition attenuates axonal degeneration, increases neuronal survival, and fosters axonal regeneration after axonal injury via a molecular switch to axon-protective, pro-survival, and growth-promoting pathways. Figure created with BioRender.com.

Recently, we have demonstrated that ULK1.DN-mediated ULK1 inhibition elicits neuroprotective effects in the CNS employing multiple models of axonal degeneration^13,14^. In the present study, we used an AAV vector to express a dominant-negative of ULK1 (ULK1.DN) and investigated its effects in different models of axonal regeneration in vitro and in vivo. We demonstrate that AAV.ULK1.DN promotes axonal regeneration, fosters neurite outgrowth and rescues CSPG-induced inhibition of neurite outgrowth in primary cortical neurons in vitro. Furthermore, we show that AAV.ULK1.DN increases neuronal survival after optic nerve transection, enhances axonal regeneration after ONC, and promotes long-term axonal protection after SCI in vivo. Interestingly, AAV.ULK1.DN also increases axonal sprouting of TH and 5-HT fibers after SCI. Finally, we show that these effects are associated with enhanced ERK1 activation and reduced expression of RhoA and ROCK2 by ULK1.DN (Fig. 8). These results therefore extend our previous studies examining the role of ULK1 in neurodegeneration^13,14^, and uncover additional pro-survival and pro-regenerative effects of ULK1 inhibition on injured axons.

Whereas our previous work has demonstrated that ULK1.DN protects axons from degeneration, it remained unknown whether ULK1.DN would also facilitate axonal regeneration. To answer this question, we first cultured primary embryonic cortical neurons in a microfluidic chamber system that separates axons from somata and dendrites, allowing us to perform selective axonal lesions and monitor axonal regeneration after axotomy in vitro by live imaging. We demonstrated that ULK1.DN enhances axonal re-growth at different time points up to 96 h after axotomy. Specifically, we found an up to sevenfold increase in the number of regenerating axons by ULK1.DN, with axons reaching 2.5 times longer distances compared to the control group. In addition, we observed a corresponding increase in neurite outgrowth for cells grown on the permissive substrate laminin after transduction with ULK1.DN. Mechanistically, we already demonstrated in our previous study that ULK1.DN inhibits autophagy, leads to an mTOR-mediated increase in translational proteins, and mediates the differential splicing of degeneration- and regeneration-associated genes^14^. Here, we additionally connect its pro-regenerative effects to increased ERK1 activation (Fig. 8), which is independent of the downstream targets CREB and ELK. The ERK1/2 pathway regulates different cellular events, such as proliferation, differentiation, and survival^31^, and has been implicated in the promotion of axonal regeneration, both in the central and peripheral nervous system^32–34^. The finding of increased ERK1 activity also corresponds well to the elevation of mTOR-dependent translation observed after transduction with AAV.ULK1.DN and SBI-0206965-mediated ULK1 inhibition in our previous studies^13,14^. mTOR-dependent translation is a key requirement for enhanced neurite outgrowth, and there is considerable crosstalk and convergence between mTOR and ERK signaling, resulting in growth promotion^35^. Therefore, our data suggest that ULK1.DN improves the intrinsic neuronal capacity for axon outgrowth and regeneration in vitro via upregulation of multiple axonal growth-associated pathways.

We have previously demonstrated that ULK1.DN modulates the differential splicing of *Kif1b* and *Ddit3*^14^. We have now characterized the protein expression of KIF1B and DDIT3 to evaluate if the differential splicing of these genes might result in detectable differences at the protein level. However, we did not find any differences in KIF1B or DDIT3 expression in AAV.ULK1.DN-transduced neurons compared with the control vector. The differentially expressed exon in *Kif1b* encodes for a sequence in the N-terminal region of the protein^14^. However, the commercially available KIF1B antibodies recognize the C-terminal region, which is not differentially spliced by ULK1.DN. Similarly, the mRNA arising from the differentially spliced exon in *Ddit3* is too similar in length compared to the full length mRNA (ten nucleotides difference)^14^. Thus, our analysis might not be able to detect these differences, indicating that additional experiments are needed to answer these questions.

To evaluate whether overexpression of ULK1.DN is also able to overcome inhibitory environmental cues, we exposed neurons transduced with AAV.ULK1.DN to the growth-inhibitory matrix CSPG. Indeed, ULK1.DN rescued CSPG-induced inhibition of neurite outgrowth. Interestingly, this is in line with decreased levels of RhoA and ROCK2 after ULK1.DN expression (Fig. 8). CSPG is known to activate the RhoA/ROCK2 pathway, resulting in impaired growth and the promotion of axonal degeneration^29,36^. In contrast, RhoA/ROCK2 downregulation and inhibition foster axonal regeneration and growth, and counteract CSPG signaling^29,36–38^. Interestingly, we already detected modulated splicing of *Ptprf*, a receptor for CSPGs that restricts axonal regeneration^39^, in ULK1.DN-transduced neurons in our previous study^14^ (Fig. 8). Thus, our data indicate that ULK1.DN additionally improves axonal outgrowth and regeneration in vitro by offsetting inhibitory environmental cues. Intriguingly, it was previously demonstrated that siRNA-mediated inhibition of ATG7, and consequently autophagy, led to increased axon elongation via reduced RhoA expression^40^, suggesting that autophagy regulates the expression of proteins that control axon outgrowth. In the present study, we demonstrate that modulation of the RhoA/ROCK2 pathway is a downstream effector mechanism of ULK1.DN-mediated ULK1 inhibition. However, we did not observe significant differences in actin dynamics in non-lesioned conditions, suggesting that ULK1.DN might influence actin dynamics only in lesioned neurons. Moreover, we found that ROCK2 or ERK expression do not change after autophagy induction using rapamycin treatment, indicating that ULK1.DN modulates these outgrowth-associated kinases independent of its autophagy-inhibiting effect.

Given that in vitro models do not fully replicate the events after injury occurring in the mammalian CNS, we evaluated the neuroprotective and regenerative effects of ULK1.DN after rat ONC. Corroborrating our results obtained in vitro, we showed that transduction of RGC with AAV.ULK1.DN increases regenerative axon growth 4 weeks after ONC. However, this effect was significant only at short distances from the lesion site, thereby resembling the positive effect on axonal regeneration observed after calcium channel inhibitor-mediated axonal stabilization after ONC in our previous study^15^. In line with this, we demonstrated attenuated acute axonal degeneration harnessing both AAV.ULK1.DN-mediated ULK1 inhibition and administration of the ULK1 inhibitor SBI-0206965 after ONC in our previous study^14^. It is, therefore, possible that the regenerative axon growth mediated by AAV.ULK1.DN after ONC is primarily based on a stabilizing effect on axons after injury rather than a substantially increased intrinsic growth capacity. A more pronounced regenerative response in vivo could thus require additional pro-regenerative signals to achieve an effect of similar strength as observed in our in vitro paradigms.

Considering the modulation of cell survival-associated proteins observed in this study, we additionally assessed cell survival in a rat model of optic nerve lesion. We found a significant increase in cell survival of axotomized RGC expressing ULK1.DN compared to control. Previously, we showed that AAV.ULK1.DN does not affect cell survival in cortical neurons in vitro^14^; however, in an MPTP-induced degeneration mouse model of Parkinson’s disease, expression of ULK1.DN increased survival of dopaminergic nigral neurons^13^. Taken together, these findings suggest that ULK1.DN-mediated ULK1 inhibition has pro-survival effects in vivo and represents a promising strategy to promote neuroprotection of RGC.

To characterize the effects of ULK1.DN on axonal regeneration in an even more complex regeneration model in vivo, we used a SCI wire-knife transection model to evaluate a potential pro-regenerative effect on CST axons. First, we demonstrated that CST axons rostral to the lesion are protected by ULK1.DN, even 5 weeks after lesion. We showed previously that ULK1.DN protects rubrospinal axons from degeneration 1 week after spinal cord lesion^14^. The results of both studies therefore indicate that ULK1 inhibition promotes axon stabilization of two different axonal tracts after SCI and, at least for the CST, for a prolonged period of time. Although axonal stabilization proximal to the lesion could improve the regenerative response of lesioned axons^15^, we did not detect any mCherry-positive axons crossing the lesion site or caudal to the lesion 5 weeks after SCI, suggesting that ULK1.DN does not promote axonal regeneration of the CST in this model. Axonal regeneration in the spinal cord, particularly of the CST, requires a strong and combined inactivation of inhibitory extracellular cues and activation of intrinsic growth signals^41^. Thus, ULK1.DN overexpression alone appears to be insufficient to induce regenerative axonal growth in the spinal cord, suggesting that combinatorial therapeutic approaches would be required to boost regeneration beyond axon protection alone^42^.

Lastly, we evaluated axon sprouting proximal to the lesion site in the SCI model. Axon sprouting has been recognized as an important mechanism contributing to functional recovery after SCI^23–25,43^. No difference in CST axon sprouting, however, could be found after transduction with ULK1.DN compared to control. Intriguingly, we showed that ULK1.DN induces rostral sprouting of 5-HT and TH axons after SCI. The increase in serotonergic and dopaminergic axon sprouting could be explained by an indirect effect of ULK1.DN expressed in CST axons, since CST axons remain more stable in ULK1.DN expressing animals and thus represent sprouting targets for 5-HT and TH axons. More detailed analyses are required, however, to dissect the precise mechanisms by which ULK1.DN influences the sprouting of 5-HT and TH axons after SCI.

Taken together, our data show that ULK1.DN promotes axonal regeneration in vitro, and enhances neuron survival and axonal regeneration in the optic nerve in vivo. In the spinal cord, ULK1.DN leads to increased axon sprouting and prolonged axonal protection. These effects are accompanied by ERK1 activation and downregulation of RhoA and ROCK2. Therefore, we hypothesize that ULK1 is a key protein regulating axonal biology, defining it as a promising molecular target to promote neuroprotection and regeneration in the CNS.

## Materials and methods

### Cloning and production of adeno-associated viral vectors

pAAV-hSyn-ULK1.DN, expressing the C-terminal domain of ULK1 (amino acids 829 to 1051) connected to an N-terminal myc-tag^44^, and pAAV-hSyn-mCherry [Genbank ID: KT345943] were cloned and produced as described previously^13,14^. Both plasmids contain two human synapsin (hSyn) promoters, one directing the expression of the reporter gene mCherry, and the second directing the expression of either the dominant-negative form of ULK1 in the vector AAV.ULK1.DN or of a non-coding transcript^16^ in the control vector AAV.CTRL, respectively. All plasmids were sequenced to confirm their correct identity.

For all experiments, AAV pseudotype 1/2 was used, which consists of an AAV2-derived genome, packed into hybrid capsids of AAV1 and a mutated form of the AAV2 capsid^45^. Generation of AAV was performed as described previously^13,14,46^. The virus stocks were tested for transduction efficiency and toxicity on primary cortical neurons; viral titers were determined with quantitative PCR.

### Neuronal cell culture and viral transduction

Primary cortical neurons were prepared from embryonic day 18 (E18) rats as described previously^14,47^. In brief, dissected embryonic cortices were trypsinized (Sigma-Aldrich) at 37 °C for 12 min, triturated, and seeded in 24-well plates pre-coated with poly-l-ornithine and laminin (both Sigma-Aldrich). In all, 4 × 10^5^ cortical neurons per well were cultured in cortex medium composed of serum-free neurobasal medium supplemented with B-27, l-glutamine, penicillin/streptomycin/neomycin (all ThermoFisher Scientific), and transferrin (AppliChem) at 37 °C and 5% CO_2_. On day in vitro (DIV) 1, the cells were transduced with AAV.CTRL (5 × 10^6^ transducing units (TU)) or AAV.ULK1.DN (9 × 10^6^ TU), resulting in only minor toxicity and equal transduction rates (70–80%). Medium changes were then performed every other day. In order to induce autophagy in selected conditions, rapamycin (750 nM, Sigma-Aldrich) was added to the medium 24 h before lysis.

### Cell lysis

Cell lysis for western blot analyses was performed using ice-cold lysis buffer composed of 0.5% (v/v) Nonidet P-40, 20 mM HEPES, 300 mM NaCl (all AppliChem), 5 mM EDTA, 1 mM dithiothreitol (both Sigma-Aldrich), plus protease inhibitor (cOmplete™) and phosphatase inhibitor (PhosSTOP™, both Roche) on DIV 8. Protein lysates were homogenized using ultrasound sonication. After centrifugation at 4 °C and 14.0 rpm for 30 min, the protein content of each sample was determined using Pierce^TM^ bicinchoninic acid assay kit (ThermoFisher Scientific) measured by a microplate reader (Spark^TM^ 10 M, Tecan). For the analysis of actin dynamics, we used a G-actin/F-actin In Vivo Assay Kit (Cytoskeleton Inc) according to the manufacturer’s instructions.

### Western blot analysis

Equal amounts of protein (10–30 µg) were loaded onto custom-made gels and separated by sodium dodecyl sulfate polyacrylamide gel electrophoresis (SDS-PAGE). Hereafter, proteins were transferred to nitrocellulose (AppliChem) or polyvinylidene difluoride membranes (GE Healthcare Life Sciences) at room temperature (RT) for 2 h or at 4 °C overnight (ON). After blocking with 5% milk or 5% bovine serum albumin (BSA) in Tris-buffered saline/0.1% Tween-20 (TBS-T, all Applichem) at RT for 1 h, membranes were incubated with primary antibodies diluted in 5% milk or 5% BSA in TBS-T at 4 °C ON. The following primary antibodies were employed: mouse *anti*-phospho-c-Jun amino-terminal kinase (*anti*-p-JNK, 1:500, 9255), rabbit *anti*-p44/42 mitogen-activated protein kinase (*anti*-Erk1/2, 1:2000, 9102), mouse *anti*-phospho-Erk1/2 (*anti*-p-Erk1/2, 1:2000, 9106), rabbit *anti*-Akt (1:1000, 9272), rabbit *anti*-collapsin response mediator protein 2 (*anti*-CRMP2, 9393, 1:1000), rabbit *anti*-phosphatase and tensin homolog (*anti*-PTEN, 1:1000, 9559), rabbit *anti*-phospho-Akt (*anti*-p-AKT, 1:1000, 9271), rabbit *anti*-phospho-glycogen synthase kinase 3 beta (*anti*-p-GSK3β, 1:1000, 5558), rabbit *anti*-phospho-PTEN (*anti*-p-PTEN, 1:1000, 9554), rabbit *anti*-phospho-signal transducer and activator of transcription 3 (*anti*-p-STAT3, 1:2000, 9145, all Cell Signaling Technology), mouse *anti*-glyceraldehyde 3-phosphate dehydrogenase (*anti*-GAPDH, 1:5000, 5G4, Hytest Ltd.), mouse *anti*-GSK3β (1:1000, 610201, BD), rabbit *anti*-phospho-CRMP2 (*anti*-p-CRMP2, 1:1000, CP2251, ECM Bioscience), mouse *anti*-RhoA (1:100, sc-418), goat *anti*-Rho-associated protein kinase 2 (*anti*-ROCK2, 1:200, sc-1851), rabbit *anti*-STAT3 (1:2000, sc-482), rabbit *anti*-CREB, (1:100, sc-186), rabbit *anti*-phospho-CREB, (1:100, sc-101663, all Santa Cruz), rabbit *anti*-DDIT3 (1:500, 15204-1-AP), rabbit *anti*-KIF1B (1:500, 15263-1-AP, both Proteintech), rabbit *anti*-Actin (1:1000, ab8227), rabbit *anti*-ELK, (1:500, ab32106, both Abcam), mouse *anti*-phospho-ELK, (1:500, MA515225, Invitrogen), mouse *anti*-Actin (1:1000, A1978, Sigma). This was followed by incubation with corresponding horseradish peroxidase (HRP)-coupled secondary antibodies at RT for 1 h. The following HRP-coupled secondary antibodies were used: horse *anti*-mouse HRP (1:1000, 7076P2), goat *anti*-rabbit HRP (1:1000, 7074P2, both Cell Signaling Technology), donkey *anti*-goat HRP (1:1000, sc-2020, Santa Cruz). After application of enhanced chemiluminescence solution composed of 90 mM p-coumaric acid, 1 M Tris pH 8.5 (both AppliChem), 250 mM luminol (Merck), and 30% hydrogen peroxide (Sigma-Aldrich), signal detection was performed within the linear range on X-Ray films (GE Healthcare Life Sciences) developed in a Curix 60 Developer. Band intensities were quantified using ImageJ (v1.50i) software. Target protein band intensities were normalized to the band intensities of the housekeeping gene GAPDH.

### Microfluidic chambers and axotomy in vitro

Microfluidic chambers^17,18^ were produced and prepared as reported previously^14,47^. Briefly described, poly(dimethylsiloxane) prepolymer and cross-linker (Sylgard 184 silicone elastomer kit, Dow Corning) were mixed, poured onto a master mold (fabricated by photolithograpy) and cured at 60 °C for 90 min. The cured piece was cut, thoroughly cleaned, sterilized using 70% ethanol and left to dry in a laminar flow hood. Glass coverslips (ThermoFisher Scientific) were flame sterilized and coated with poly-d-lysine (Sigma-Aldrich). Chambers were then mounted onto the coverslip. In all, 2.1 × 10^5^ rat cortical neurons were prepared as described above, resuspended in cortex medium and seeded into one main channel of the microfluidic culture system. 4 h later, transduction with AAV vectors (AAV.CTRL: 1.5 × 10^6^ TU; AAV.ULK1.DN: 4 × 10^6^ TU) was performed. Changes of half of the total medium volume were then performed on both sides of the chamber every to every other day, maintaining a higher medium volume on the cell side to direct axonal growth through the microgrooves. After 7–9 DIV, axons reached the other main channel of the chamber system. Using gentle vacuum suction in this “axonal compartment”, an axotomy was performed to induce axonal degeneration. Afterwards, the channel was refilled with fresh cortex medium.

### Live-imaging and quantification of axonal regeneration in vitro

For live imaging of axonal regeneration in microfluidic chambers, mCherry-labeled axons were imaged in a microscope incubation system (Leica DMI6000B, x16 magnification, 37 °C, 5% CO_2_) equipped with Leica Application Suite software. Axons were imaged directly before and at 5 min, 24, 48, 72, and 96 h after axotomy. Only areas with visually confirmed axonal lesion as compared to the images taken directly before axotomy were taken into account for evaluation. Using a superimposed counting grid in the Leica Application Suite software, the number of regenerating axons was quantified for 10 microgrooves in the middle of each chamber at defined distances (100, 200, 400, 600, 800, 900, 1000 µm) from the lesion site for each time point in a blinded fashion. To account for slightly varying axon numbers in the different chambers, the number of regenerating axons at each time point was normalized to the number of axons at the distal aperture of the microgrooves before axotomy for each chamber.

### Neurite outgrowth experiments and quantification

For neurite outgrowth experiments, 24-well-plates were coated with poly-l-ornithine (Sigma-Aldrich) and either laminin (Sigma-Aldrich) or CSPG (1.25 µg/mL, Millipore). In all, 2 × 10^4^ cortical neurons were prepared, plated in cortex medium and cultured as described above. Four hours after seeding, cells were transduced with AAV.CTRL or AAV.ULK1.DN (both 2 × 10^6^ TU per well). Medium changes were performed every other day. On DIV 7, photomicrographs of 5 × 5 visual fields (each of 800 x 600 µm length) were taken in the center of each well using a microscope incubation system (Leica DMI6000B, x16 magnification, 37 °C, 5% CO_2_) equipped with Leica Application Suite software. Employing the neurite tracing plugin for ImageJ NeuronJ (v1.4.3)^48^, the total neurite length was quantified on two view fields and then divided by the manually counted number of cells to determine the total neurite length per cell. Two to three wells were analyzed for each condition for each experiment in a blinded fashion. To account for variability in the absolute neurite length between independent cultures, the absolute neurite length per cell in each condition in each independent experiment was normalized to the absolute neurite length per cell of the laminin-coated control condition transduced with AAV.CTRL.

### Animal experiments

Adult female Wistar rats weighing 200–300 g were used for all in vivo experiments. Animals were maintained at 12/12 h light and dark cycle with free access to food and water. The animals were randomly allocated to each group. All animal experiments were performed with the approval of the governmental authorities and according to the legislation of the local animal research council of the State of Lower Saxony (Braunschweig) and the State Office, Environmental and Consumer Protection of North Rhine-Westphalia, LANUV NRW (Recklinghausen), both in Germany, and the Ethics Committee on the Use of Animals in Scientific Experimentation from the Federal University of Minas Gerais, Brazil.

### Intravitreal virus injection

All procedures involving optic nerve lesion experiments (intravitreal virus injection, optic nerve transection and optic nerve crush) were performed under deep anesthesia with 10% ketamine (95 mg/kg body weight) and 2% xylazine (7 mg/kg body weight) injected intraperitoneally.

To transduce retinal ganglion cells (RGC), 21 days prior to optic nerve transection or crush, intravitreal injection of AAV.CTRL (1 × 10^8^ TU) or AAV.ULK1.DN (2 × 10^8^ TU) in a volume of 4 µl was performed using a Hamilton syringe (701RN, 30 s gauge, Hamilton). These viral titers were used because they were tested previously and result in similar transduction rates of RGC, which were more concentrated in the center of the retina. Animals with low virus transduction (<1/3 retinal area), verified by analyzing mCherry-positive RGCs in retina flat-mount, were excluded.

### Optic nerve transection and retrograde labeling of RGCs

To evaluate RGC survival, an optic nerve transection was performed according to a previously published protocol^21^. Under deep anesthesia, the skin of the animal was incised close to the orbital rim, the orbita was opened, and the lacrimal gland was moved to the rostral side of the orbita. The superior rectus muscle was detached from its insertion point and the eye bulb was rotated in ventral direction. The optic nerve was exposed by a longitudinal incision of the optic nerve sheath. Then, using a pair of microscissors, the optic nerve was completely transected at ~2 mm distance from the eye bulb. A 1 × 1 mm piece of gel foam (Technew) was soaked in 2 µl of FluoroGold 4% (Hydroxystilbamidine, Life Technologies) and placed onto the optic nerve stump in order to retrogradely label RGC. The exposed skull was sutured, and rats were allowed to wake up from anesthesia.

### Optic nerve crush

For the evaluation of axonal regeneration in the optic nerve, an ONC was performed on the left eye as described previously^20^. Under deep anesthesia, the optic nerve was exposed as described above. Care was taken not to damage the central retinal artery. For the crush lesion, a 10-0 polyamide suture (Ethicon) was tightly constricted around the optic nerve at a distance of ~2 mm from the eye bulb for a duration of 30 s. Then, the suture was removed, all tissue put back in situ, the skin was sutured, and the animals allowed to wake up from anesthesia.

### Perfusion, retina flat-mount, and optic nerve cryosectioning

Fourteen days after optic nerve transection or 28 days after ONC, the animals were perfused transcardially with 250 ml of phosphate-buffered saline (PBS–pH 7.4) followed by 250 ml 4% paraformaldehyde (PFA, Merck) in PBS. The optic nerve and eye bulb were removed en bloc and post-fixed in 4% PFA for 2 h. For FluoroGold-positive RGC counting, the retina was dissected, flat-mounted in 50% glycerol in PBS on glass slides and stored at 4 °C until further quantification. For the quantification of axonal regeneration, the optic nerves were dissected containing the proximal and distal side of the crush lesion, and incubated in 30% sucrose (Sigma-Aldrich) for 48 h. Longitudinal cryosections (16 µm) were generated using a cryostat (Leica), collected on superfrost slides (Fischer Scientific), and stored at –20 °C until further evaluation.

### Quantification of RGC survival

For quantification of RGC survival, retinal flat-mounts were imaged using a fluorescence microscope (Axio Imager Z2–Apotome 2) equipped with ZEN Software (both Zeiss) using a UV filter (365/420 nm). Images were taken in the central region of the retina, where virus transduction was concentrated, using a 20x objective (0–1 mm from the optic nerve head along the retina diameter; four images per retina). RGCs were discriminated from other FluoroGold-positive cells (microglia, macrophages, etc.) by morphological criteria (soma size, form of processes) and counted in a blinded fashion.

### Quantification of axonal regeneration in the optic nerve

For the quantification of RGC axonal regeneration, slides containing optic nerve longitudinal sections (16 µm) were immunostained for GAP43. Photomicrographs were taken using a fluorescence microscope (Axio Imager Z2–Apotome 2) equipped with ZEN Software (both Zeiss). The number of regenerating GAP43-positive axons was counted, in a blinded fashion, at defined distances distal to the crush site using a counting grid superimposed on the photomicrograph.

### Stereotactic viral injection into the sensorimotor cortex and spinal cord injury

Animals were anesthetized using 2–3% isoflurane in O_2_ and NO_2_ at a ratio of 1:2 as described before^37^. Two small holes were drilled 0.2 mm posterior to bregma and 0.24 mm lateral to the midline on both sides, then, 2 µl of AAV.CTRL (1 × 10^8^ TU) and AAV.ULK1.DN (5 × 10^8^ TU) were injected into the respective sensorimotor cortex to label both CSTs. Spinal cord surgery including transection of the dorsal CST was performed 3 weeks after AAV injections as described before^22^. Briefly, the dura was opened at thoracic level 8 (Th8), and the dorsal CST and dorsal columns were transected using a Scouten wire knife (Bilaney). Following surgery, the overlying tissues were sutured in layers, and the animals were housed with food and water ad libitum and were allowed to recover. Post operative care included prophylactic daily oral Baytril™ (Bayer Health Care) administration for 1 week.

Five weeks after SCI, animals were transcardially perfused with 4% PFA in PBS. Then, the spinal cord was removed, post-fixed in 4% PFA, cryoprotected in sucrose (30%) for 3 days, and stored at −80 °C. Next, parasagittal cryosections (18 μm thick slices) including the lesion site were prepared from spinal cord samples using a cryostat (Leica), collected on superfrost slides (Fischer Scientific) and stored at −20 °C until immunostaining.

### Quantification of spinal cord lesion size

Parasagittal sections including the lesion site were imaged on an Axioplan microscope equipped with a MosaiX software module (Zeiss). The lesion area, indicated by no GFAP-immunoreactivity, was surrounded with the area-measurement-tool of Zeiss Axiovision software, the maximum area of the lesion was quantified in µm^2^ in three adjacent sections and compared between the treatment groups.

### Quantification of axonal degeneration rostral to the lesion in the spinal cord

Parasagittal sections including the lesion site and rostral and cauldal regions were imaged as described above and axons expressing mCherry were quantified in a region up to 2 mm rostral to the lesion site. mCherry-positive axons were quantified in three sections per animal and normalized to the number of transduced axons quantified in coronal sections at cervical level C2. The number of axons was counted at different distances (50, 100, 200, 400, 600, 800, 1000, and 2000 µm) from the lesion border. The number of axons was then compared between the treatment groups.

### Quantification of axon sprouting in the spinal cord

Assessment of rostral sprouting of mCherry-, 5-HT- and TH-labeled axons was performed as described before^26^. Briefly, images of the white matter near the intermediolateral column were captured for all analyses and, for 5-HT- and TH- axonal sprouting, images of the central canal, dorsal horn, and ventral horn were acquired additionally, all using coronal sections at cervical level C2. Images were analyzed using the ImageJ “Feature J” plugin, by converting the original image files to 8-bit before the application of the “smallest Hessian” eigenvalue was performed^49^. The resulting images were then converted to a binary image, where mCherry-, 5-HT- or TH-positive axons were threshold-adjusted. For the above-mentioned spinal cord areas, the relative area occupied by mCherry-, 5-HT- or TH-positive axons was determined and compared between the treatment groups.

### Immunohistochemical stainings

Immunostaining was performed on optic nerve and spinal cord sections. An antibody against growth-associated protein-43 (rabbit anti-GAP43, 1:250, ab16053, Abcam) was used to visualize regenerating axons in the optic nerve. Antibodies against glial fibrillary acidic protein (rabbit anti-GFAP, 1:500, Z0334, Dako), tyrosine hydroxylase (rabbit anti-TH, 1:1000, 220-0694, Zytomed), and serotonin (rabbit anti-5HT, 1:30, CAF10367, Biozol) were used for the staining of spinal cord sections to visualize the lesion area and sprouting of TH- and 5-HT-labeled axons, respectively.

For optic nerve immunostaining, sections were permeabilized with 0.3% Triton X-100 and 0.05% Tween-20 in PBS for 15 min and incubated with the blocking solution 10% normal goat serum (NGS, Sigma-Aldrich) in PBS at RT. Then, the sections were incubated ON at 4 °C with anti-GAP43 primary antibody diluted in the same blocking solution. Sections were incubated with secondary antibody Alexa-488 anti-rabbit (1:1000, ThermoFisher Scientific) for 1 h at RT. The sections were counter-stained with 4,6-diamidino-2-phenylindole (DAPI) and mounted in Mowiol (both Sigma-Aldrich).

For spinal cord immunostaining, sections were rehydrated in PBS for 15 min, followed by 5 min incubation in 0.3% Sudan black to reduce potential background staining. Sections were blocked in 10% NGS/PBS at RT. Primary antibodies were incubated in 1% NGS/PBS ON at 4 °C. Respective sections were incubated with secondary antibody Alexa-488 anti-rabbit for 1 h at RT. Then, sections were counter-stained with DAPI and mounted with Mowiol.

### Data analysis

Experiments performed in this study were similar to those routinely done in our labs. Therefore, the sample size for all experiments (cell culture, spinal cord injury and optic nerve crush) were similar to other experiments routinely performed in our labs and no statistical methods were used to additionally predetermine sample sizes. Data distribution was assumed to be normal and variances were assumed to be similar, without formal testing. Statistical analyses were conducted using Prism 7 software (GraphPad Software). Comparisons of two groups were done by two-tailed unpaired *t*-test and multiple group comparisons by one-way analysis of variance (ANOVA) with Tukey’s post hoc test. The statistical test and number of in vitro or in vivo experiments used for each analysis are indicated in each figure legend. Data are presented as lines or bars with single data points and means ± SEM. Differences were considered significant when *P* < 0.05 (*P < 0.05; **P < 0.01; ***P < 0.001; ns: not significant).

## Supplementary information

SUPPLEMENTAL FIGURE LEGEND

Figure S1

Figure S2

Figure S3

Figure S4

Figure S5
