# Supplementary material for: AAV-mediated inhibition of ULK1 promotes axonal regeneration in the central nervous system in vitro and in vivo
Source: Cell Death Dis. 2021 Feb 26;12(2):213. doi: 10.1038/s41419-021-03503-3 (PMC7910615; doi:10.1038/s41419-021-03503-3)
Supplement: Supplementary file 1 — SUPPLEMENTAL FIGURE LEGEND [file 41419_2021_3503_MOESM1_ESM.docx]

**SUPPLEMENTAL FIGURE LEGEND**

**Fig. S1: Equal transduction rates in the CST after transduction with AAV.CTRL and AAV.ULK1.DN. (A)** Representative images of coronal spinal cord sections at the cervical level 2 (C2) transduced with given AAV showing CST mCherry-positive axons (red) and DAPI (blue) Scale bar: 100 μm. **(B)** Quantification of the total number of labeled axons after transduction with AAV.CTRL (n=6 animals) and AAV.ULK1.DN (n=5 animals). Data are presented as single data points and means ± SEM. No significant difference, according to two-tailed unpaired t-test.

**Fig. S2: AAV.ULK1.DN does not influence lesion size after spinal cord injury. (A)** Representative images of parasagittal spinal cord sections at the cervical level 2 (C2) transduced with given AAV showing staining for GFAP (green) and DAPI (blue) Scale bar: 100 μm. **(B)** Quantification of the lesion size after transduction with AAV.CTRL (n=6 animals) and AAV.ULK1.DN (n=5 animals). Data are presented as single data points and means ± SEM. No significant difference, according to two-tailed unpaired t-test.

**Fig. S3: CST sprouting is not influenced by AAV.ULK1.DN after spinal cord injury. (A)** Representative images of coronal spinal cord sections transduced with given AAV showing mCherry-positive axons (red) and staining for DAPI (blue) Scale bar: 100 μm. **(B)** Quantification of mCherry-positive axon sprouting after transduction with AAV.CTRL (n=6 animals) and AAV.ULK1.DN (n=5 animals). AU: arbitrary units. Data are presented as single data points and means ± SEM. No significant difference, according to two-tailed unpaired t-test.

**Fig. S4: Effect of AAV.ULK1.DN on AKT, PTEN, CRMP2, DDIT3, KIF1B, and F/G actin.** Lysates were obtained from E18 rat cortical neurons on DIV 8 after transduction with AAV.ULK1.DN or AAV.mCherry. Representative immunoblots of AKT/p-AKT **(A)**, PTEN/p-PTEN **(B)**, CRMP2 **(C)**, p-CRMP2 **(D)**, DDIT3 **(E)**, KIF1B **(F)**, F/G actin **(G)** and the corresponding bands of the loading control GAPDH are shown. Quantifications of the band intensities of AKT/p-AKT **(A** - n=4/6 independent cultures**)**, PTEN/p-PTEN **(B** - n=4/6 independent cultures**)**, CRMP2 **(C** - n=6 independent cultures**)**, p-CRMP2 **(D** - n=5 independent cultures**)**, DDIT3 **(E** - n=5 independent cultures**)**, KIF1B **(F** - n=5 independent cultures**)** and F/G actin **(G** - n=3 independent cultures**)**. CTR: control. RAP: addition of rapamycin (750 nM) 24 hours before lysis. Data are presented as single data points and means ± SEM. *P<0.05, ***P<0.001, ns: no significant difference, according to one-way ANOVA and Tukey’s multiple comparisons test **(A-F)** or t-test **(G)**.

**Fig. S5: Effect of AAV.ULK1.DN on GSK3ß/p-GSK3ß, JNK, STAT/p-STAT3, CREB/p-CREB, and ELK/p-ELK.** Representative immunoblots of GSK3ß/p-GSK3ß **(A)**, p-JNK **(B)**, STAT3 **(C)**, p-STAT3 **(D),** CREB/p-CREB **(E)**, ELK/p-ELK **(F)** and the corresponding bands of the loading control GAPDH or Actin are shown. Quantifications of the band intensities of GSK3ß/p-GSK3ß **(A)**, p-JNK **(B)**, STAT3 **(C)**, p-STAT3 **(D)**, CREB/p-CREB **(E)**, ELK/p-ELK **(F)**. CTR: control. RAP: addition of rapamycin (750 nM) 24 hours before lysis. Data are presented as single data points and means ± SEM. n = 4/6 independent cultures. *P<0.05, **P<0.01, ***P<0.001, ns: no significant difference, according to one-way ANOVA and Tukey’s multiple comparisons test.
