# Supplementary figures and images for: AAV-mediated inhibition of ULK1 promotes axonal regeneration in the central nervous system in vitro and in vivo
Source: Cell Death Dis. 2021 Feb 26;12(2):213. doi: 10.1038/s41419-021-03503-3 (PMC7910615; doi:10.1038/s41419-021-03503-3)

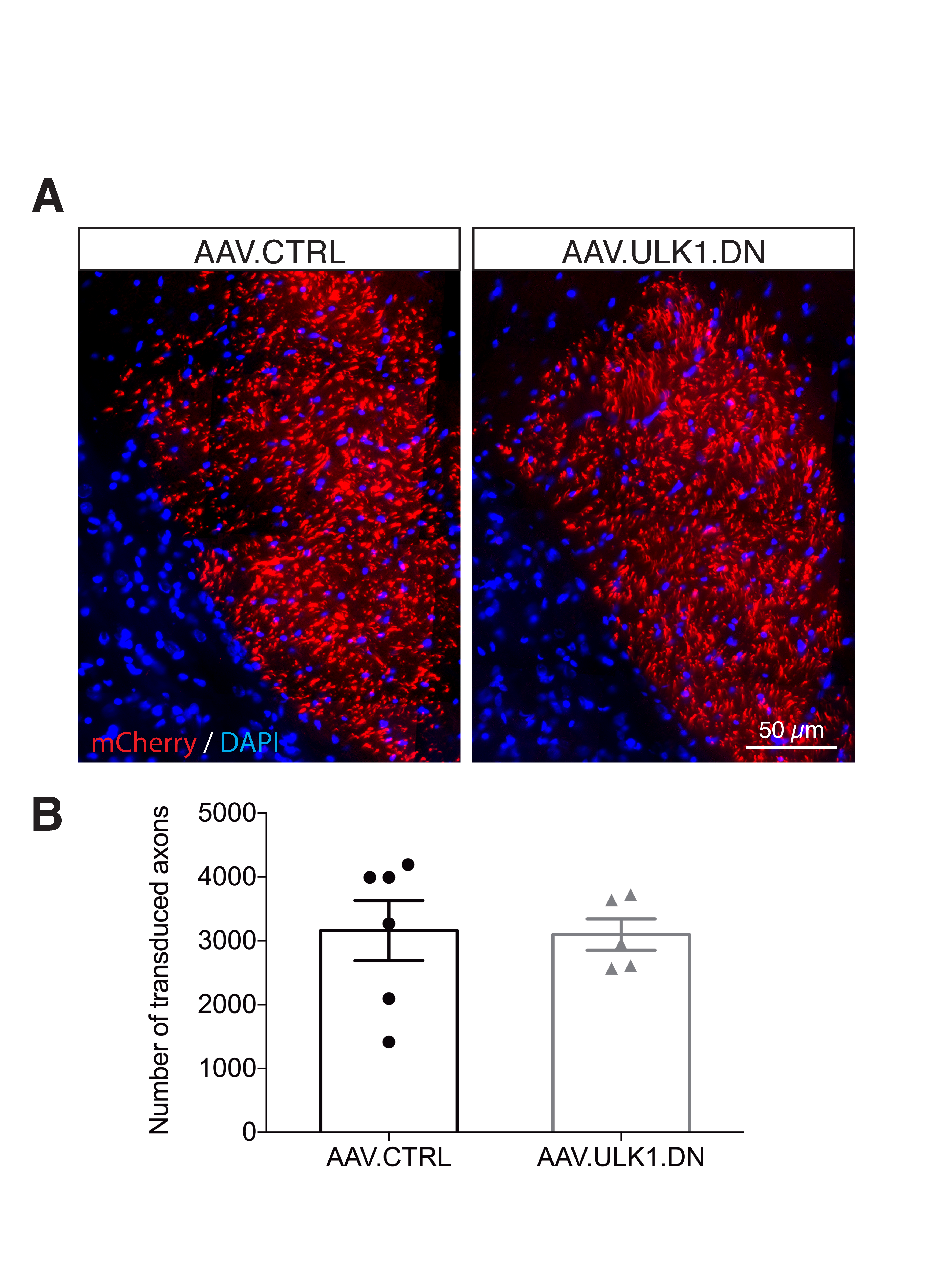

Supplement: Supplementary file 2 — Figure S1 [file 41419_2021_3503_MOESM2_ESM.tif]

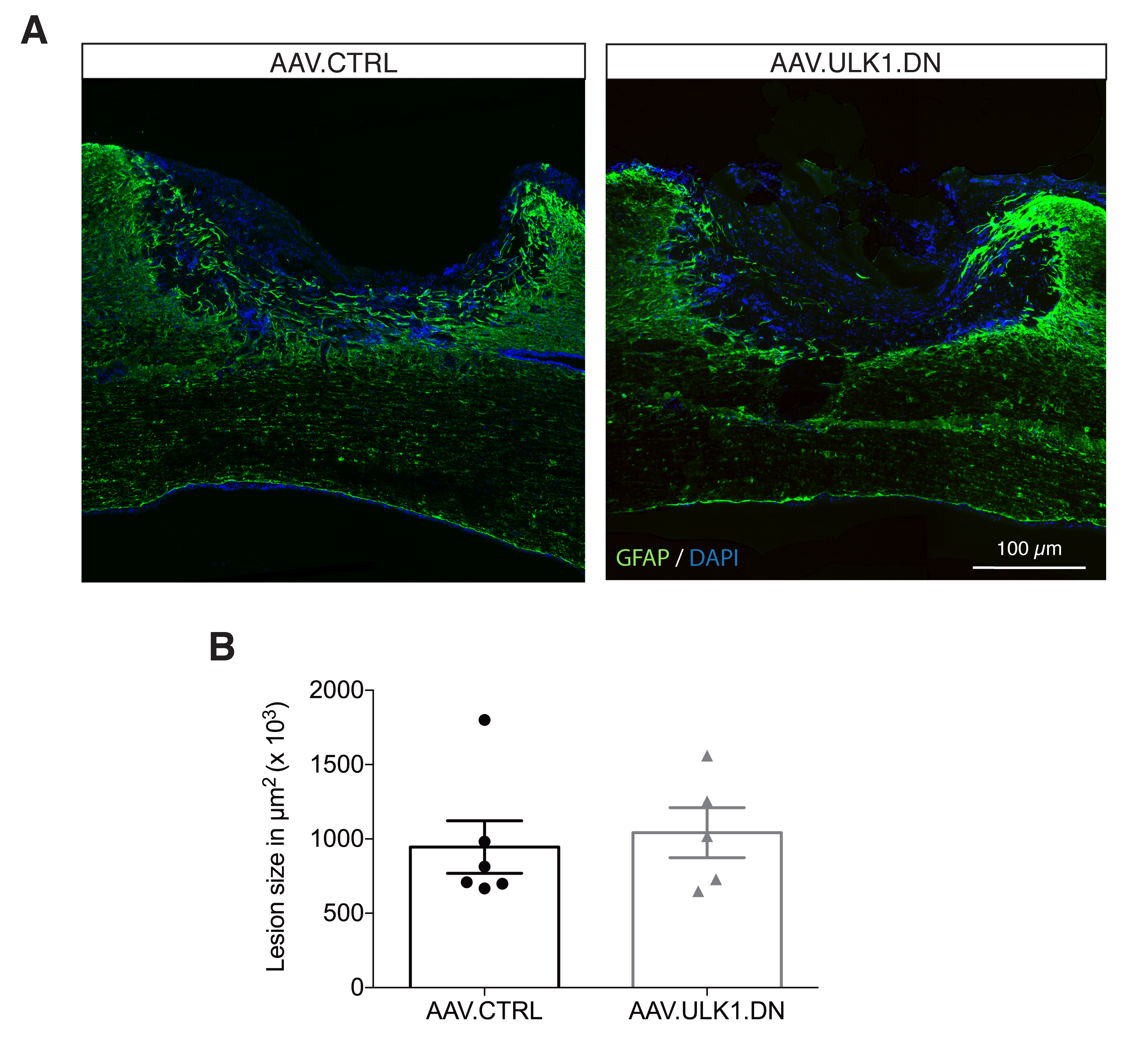

Supplement: Supplementary file 3 — Figure S2 [file 41419_2021_3503_MOESM3_ESM.tif]

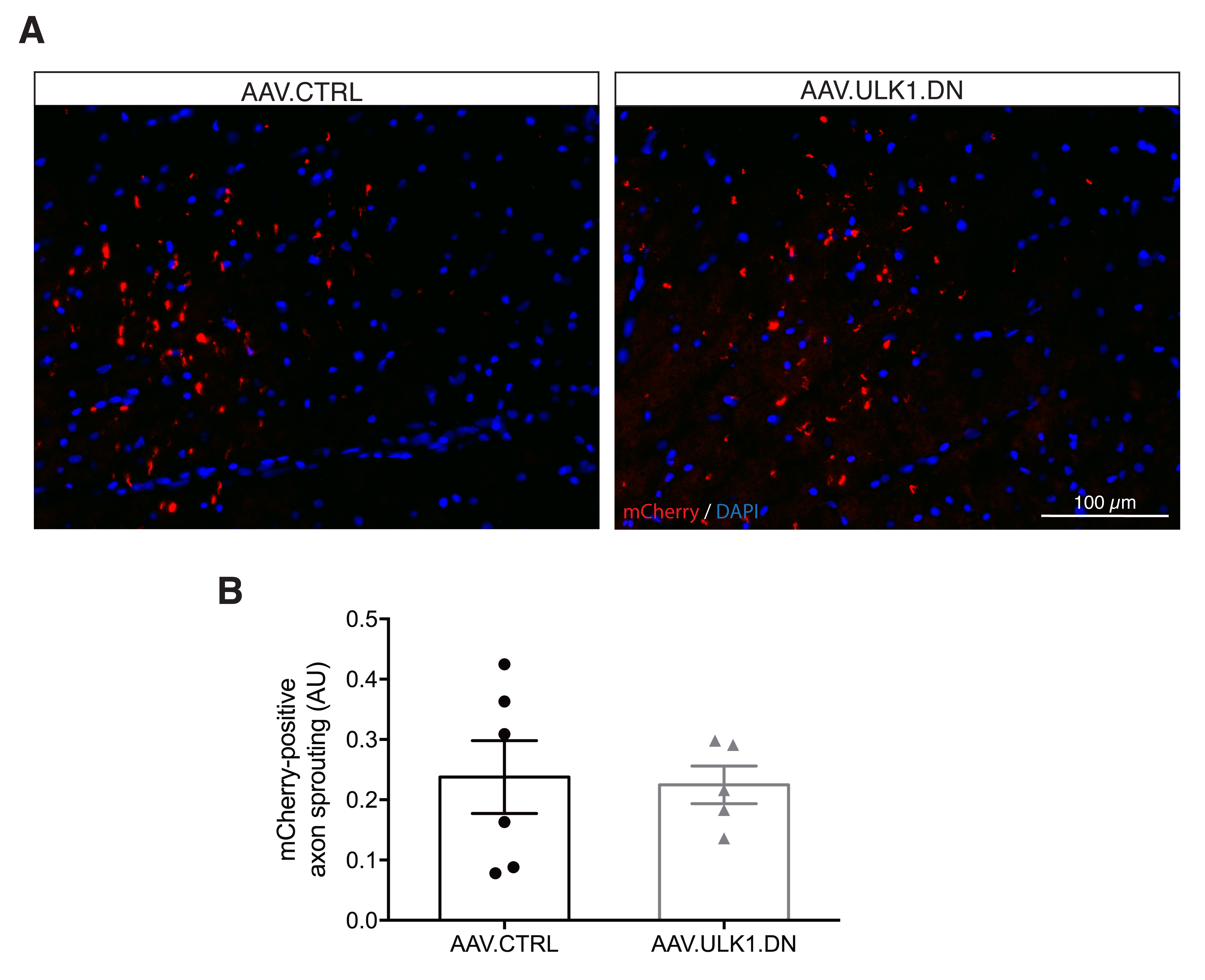

Supplement: Supplementary file 4 — Figure S3 [file 41419_2021_3503_MOESM4_ESM.tif]

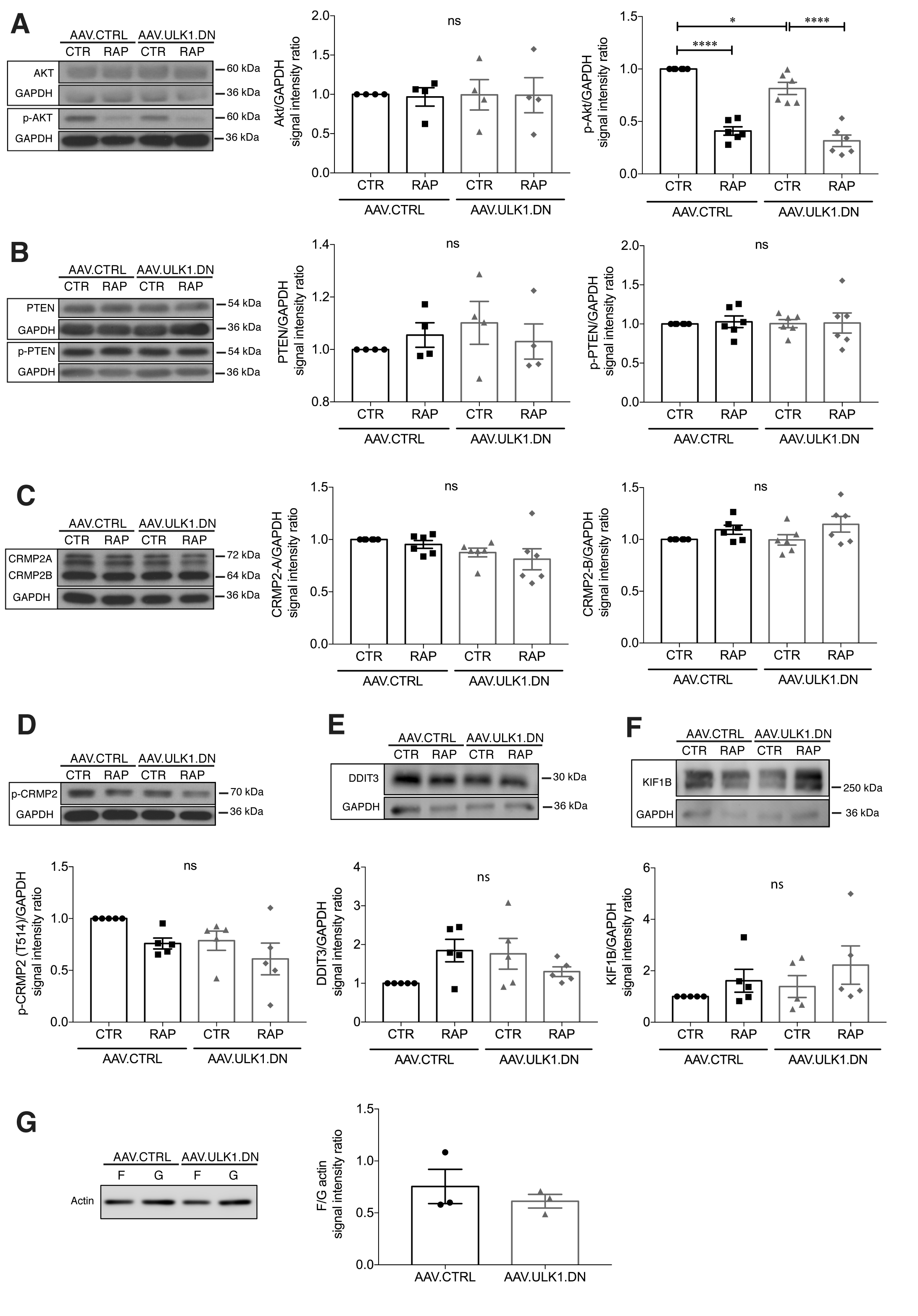

Supplement: Supplementary file 5 — Figure S4 [file 41419_2021_3503_MOESM5_ESM.tif]

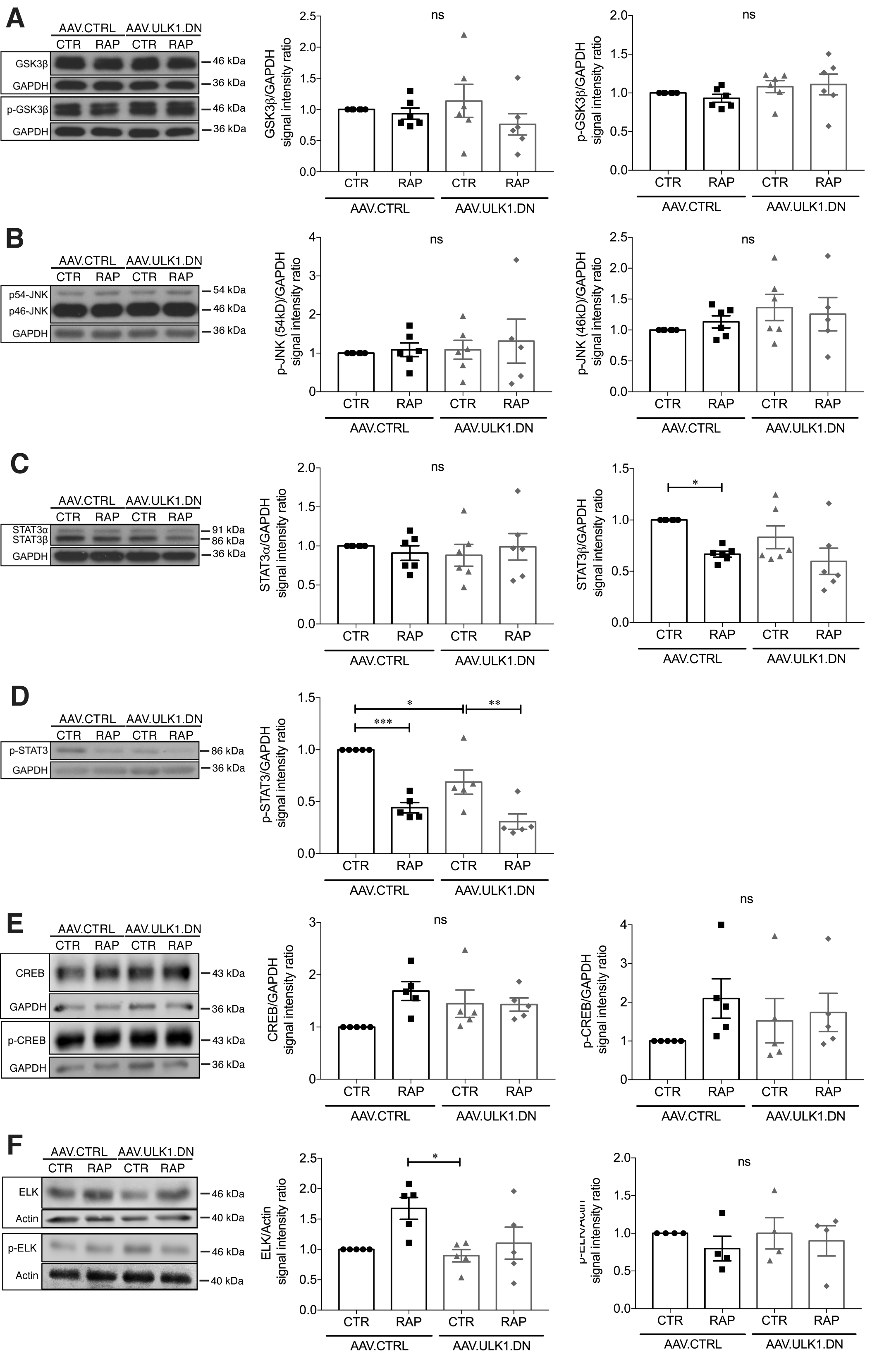

Supplement: Supplementary file 6 — Figure S5 [file 41419_2021_3503_MOESM6_ESM.tif]
